# Caffeine Taste Signaling in *Drosophila* Larvae

**DOI:** 10.3389/fncel.2016.00193

**Published:** 2016-08-09

**Authors:** Anthi A. Apostolopoulou, Saskia Köhn, Bernhard Stehle, Michael Lutz, Alexander Wüst, Lorena Mazija, Anna Rist, C. Giovanni Galizia, Alja Lüdke, Andreas S. Thum

**Affiliations:** ^1^Department of Biology, University of KonstanzKonstanz, Germany; ^2^Department of Biomedical Science, University of SheffieldSheffield, UK; ^3^Zukunftskolleg, University of KonstanzKonstanz, Germany

**Keywords:** *Drosophila* larvae, feeding, learning and memory, choice behavior, caffeine, gustatory receptor, single cell analysis

## Abstract

The *Drosophila* larva has a simple peripheral nervous system with a comparably small number of sensory neurons located externally at the head or internally along the pharynx to assess its chemical environment. It is assumed that larval taste coding occurs mainly via external organs (the dorsal, terminal, and ventral organ). However, the contribution of the internal pharyngeal sensory organs has not been explored. Here we find that larvae require a single pharyngeal gustatory receptor neuron pair called D1, which is located in the dorsal pharyngeal sensilla, in order to avoid caffeine and to associate an odor with caffeine punishment. In contrast, caffeine-driven reduction in feeding in non-choice situations does not require D1. Hence, this work provides data on taste coding via different receptor neurons, depending on the behavioral context. Furthermore, we show that the larval pharyngeal system is involved in bitter tasting. Using ectopic expressions, we show that the caffeine receptor in neuron D1 requires the function of at least four receptor genes: the putative co-receptors *Gr33a, Gr66a*, the putative caffeine-specific receptor Gr93a, and yet unknown additional molecular component(s). This suggests that larval taste perception is more complex than previously assumed already at the sensory level. Taste information from different sensory organs located outside at the head or inside along the pharynx of the larva is assembled to trigger taste guided behaviors.

## Introduction

Taste is a vital sense for animals. Sensory cells located in taste organs, such as the tongue of mammals or the proboscis of insects, are dedicated to discriminating between structurally diverse chemical compounds (reviewed in Apostolopoulou et al., 2015; Freeman and Dahanukar, 2015; French et al., 2015; Joseph and Carlson, 2015; Kikut-Ligaj and Trzcielinska-Lorych, 2015). Some of these compounds indicate the presence of nutrients in a food source, while others may even signal toxicity. In humans, harmful compounds are often perceived as bitter and induce innate aversion (Ventura and Worobey, 2013; Barretto et al., 2015). By extension, bitter sensation is inferred in animals, including insects, for substances that elicit innate aversive reactions.

Taste-driven behavior has many facets, including food-seeking or feeding-related behaviors and also communication and the identification of mating partners and predators (Bray and Amrein, 2003). Tastant valence can be concentration-dependent, as low salt concentrations are appetitive, whereas higher concentrations are aversive (Niewalda et al., 2008; Zhang et al., 2013; Alves et al., 2014). In addition, innate attraction can be reversed during the animals' life (Xu et al., 2008). Furthermore, taste-dependent behavior is state-dependent. Feeding depends on the hunger state of an animal and otherwise aversive compounds can become appealing for reasons of self-medication (Bernays and Singer, 2005; Milan et al., 2012; Abbott, 2014). Given this complexity it is not surprising that many details of taste coding such as a precise number and molecular function of sensory neurons and taste receptors or the functional dissociation between internal and external sensory organs remain to be investigated.

To learn more about bitter taste perception at the cellular and molecular level we studied caffeine sensation using *Drosophila* larvae as a model system. The *Drosophila* larva has a simple peripheral nervous system with a small number of sensory neurons to assess its chemical environment (Singh and Singh, 1984; Tissot et al., 1997; Python and Stocker, 2002; Kwon et al., 2011; Apostolopoulou et al., 2014a). Three external chemosensory organs are located at the tip of the larval head; the dorsal (DO, which mainly serves olfactory function), terminal (TO), and ventral organ (VO). Internally, three organs are located along the pharynx: the dorsal (DPS), posterior (PPS), and ventral (VPS) pharyngeal sensilla. In total these organs give rise to about 114 pairs of sensory neurons which have been suggested to function in gustation, olfaction, thermosensation, hygrosensation, and mechanosensation (Singh and Singh, 1984; Python and Stocker, 2002; Fishilevich et al., 2005; Kreher et al., 2005; Apostolopoulou et al., 2015; Klein et al., 2015; Ni et al., 2016). Thus, larvae have at least two taste subsystems, an external one and a pharyngeal one. Taste coding in the pharyngeal system was not analyzed so far.

Anatomical studies suggest that the larva perceives bitter taste by a total of 12 gustatory receptor neuron (GRN) pairs, six in the TO and six located internally along the pharynx (Kwon et al., 2011; Apostolopoulou et al., 2014a; Kim et al., 2016). Related GRNs acquire their function by the expression of different combinations of gustatory receptors (Grs). In *Drosophila* 60 gustatory receptor genes code for 68 gustatory receptors. Their majority detects bitter compounds (Clyne et al., 2000; Dunipace et al., 2001; Scott et al., 2001; Robertson et al., 2003). Although Grs in *Drosophila* share no homology to mammalian taste receptors (Robertson et al., 2003; Zhang et al., 2011), they seem to share similarities in processing the valence of bitter and sweet stimuli. Current data from adult *Drosophila* suggest that several GR proteins are needed to form a functional receptor unit (Jiao et al., 2008; Lee et al., 2009, 2010). Bitter receptors may need the co-expression of Gr32a, *Gr33a, Gr66a* (Moon et al., 2009; Lee et al., 2010) which may be bitter co-receptors (Weiss et al., 2011). Beside these co-receptors, additional receptors may have a more specific role in the detection of particular chemicals such as Gr59c for berberine, lobeline, and denatonium (Weiss et al., 2011), Gr8a for L-canavanine (Lee et al., 2012), and Gr47a for strychnine (Lee et al., 2015, but see also Delventhal and Carlson, 2016). Whether this is true for the larval stages has not been addressed systematically.

How larvae manage to sample and process a wide range of chemicals with only a few neurons that express different sets of GRs remains unknown. Of the three putative bitter co-receptors found in adults, the larva expresses only *Gr66a* and *Gr33a* in 12 GRNs (Kwon et al., 2011; Apostolopoulou et al., 2014a; Kim et al., 2016). Only some of them express Gr32a (Kwon et al., 2011). One GRN of the TO was suggested to respond to opposing tastes, such as sweet and bitter (van Giesen et al., 2016). These results suggest a complexity that is far from being understood. Therefore, more experimental work is required to understand larval bitter sensation and taste processing in general. Especially the role of the pharyngeal sensory neurons remains elusive given the lack of anatomical, molecular, and behavioral data.

Here we find that larvae require only a single pharyngeal GRN pair called D1, which is located in the DPS, in order to avoid caffeine and to associate an odor with caffeine dependent punishment. In contrast, caffeine-driven reduction in feeding in non-choice situations does not require D1. In addition, we show that the molecular mechanism which provides D1 with the ability to detect caffeine is conserved throughout metamorphosis. As in adult *Drosophila*, caffeine sensation requires *Gr33a, Gr66a*, and Gr93a receptor gene function (Lee et al., 2009). Caffeine sensation also requires an additional unknown Gr gene because co-expression of *Gr33a, Gr66a*, and Gr93a receptor genes in the pharyngeal D2 neuron pair is not sufficient to introduce caffeine sensitivity. Yet, we cannot exclude the possibility that additional Gr genes expressed in the D2 neuron pair antagonize Gr93a receptor gene function (Delventhal and Carlson, 2016). Together, this work provides—to our knowledge—the first functional study on taste coding via the larval pharyngeal system.

## Materials and methods

### Fly stocks and maintenance

Flies were maintained on standard *Drosophila* medium at 25°C. For all experiments, flies were transferred to new vials and allowed to lay eggs for 2 days. Experiments were performed 5 or 6 days after egg laying. Third instar, feeding stage larvae were used in groups of about 30 animals for behavioral experiments or individually for anatomical approaches and Ca^2+^-imaging experiments. Gr-Gal4 lines (*Gr66a-Gal4, Gr33a-Gal4, Gr10a-Gal4, Gr36c-Gal4, Gr94a-Gal4, Gr97a-Gal4, Gr57d-Gal4, Gr59d-Gal4*, and *Gr93a-Gal4*), WT CantonS, UAS-*hid,rpr*, and UAS-*mCD8::GFP* stocks were kindly provided by the Carlson, Scott, Heisenberg, Sprecher, and Tanimoto labs. UAS-*GCaMP6m* and mutants for gustatory and olfactory receptor genes (*Gr66aex83, Gr33a1*, and *Gr93a3*) were obtained from the Bloomington Stock Center (Bloomington Stock Numbers 42748, 35528, 31427, and 27592). w1118 was crossed to different lines for heterozygous controls. In addition, we used *Gr33a1; Gr66aex83* double mutant larvae, UAS-*GCaMP6m*; *Gr33a-Gal4* larvae, UAS-*GCaMP6m*; *Gr33a1* larvae, *Gr93a-Gal4*; *Gr33a1* larvae, UAS-*GCaMP6m*; *Gr93a3* larvae, and *Gr93a-Gal4*; *Gr93a3* larvae that were established by crosses using a double balancer stock (Bloomington Stock Number 3704).

### Caffeine-dependent choice behavior

Experiments were performed using standard methods (Niewalda et al., 2008; Schipanski et al., 2008; El-Keredy et al., 2012; Rohwedder et al., 2012; Apostolopoulou et al., 2014a). 1.0% (w/v) agarose solution (Sigma Aldrich Cat. No.: A5093; CAS No.: 9012-36-6) was boiled in a microwave and filled as a thin layer into Petri dishes (85 mm diameter, Cat. No.: 82.1472, Sarstedt, Nümbrecht, Germany). After cooling the agarose was removed from half of the plate. The empty half was filled by 1.0% (w/v) agarose solution in addition containing 50 mM caffeine (Sigma Aldrich cat. no.: 27600) if not indicated otherwise. For the test, 30 larvae were put in the middle of a Petri dish. Larvae were counted after 5 min as being located on either the caffeine side, the no-caffeine side, or a middle neutral side (an area of about 10 mm width running vertically in the middle of the plate).

The preference indices for choice behavior were calculated as follows:

$$\begin{array}{l} \text{Preference Index}=\frac{\left(\#\text{CAFFEINE }-\#\text{PURE AGAROSE}\right)}{\#\text{TOTAL}} \end{array}$$

Negative preference indices therefore indicate aversion to caffeine.

### Feeding

Experiments were performed using standard methods (Schipanski et al., 2008; El-Keredy et al., 2012; Rohwedder et al., 2012; Apostolopoulou et al., 2014a; König et al., 2014). For control experiments, Petri dishes were filled with a solution of 1% (w/v) agarose and 2% (w/v) indigo carmin (Sigma Aldrich cat. no.: 73436). For experimental groups, Petri dishes were filled with a solution of 1% (w/v) agarose, 2% (w/v) indigo carmin, and 50 mM caffeine. Experimental larvae were allowed to feed on dishes for 30 min. They were then washed in tap water and homogenized in 500 μl of 1 M ascorbic acid solution (Sigma Aldrich cat. no.: A7506). The homogenate was centrifuged for 5 min at 13,400 rpm and the supernatant was filtered using a syringe filter (millipore, 5 μm pores, Darmstadt, Germany) into a new Eppendorf cup. Subsequently, the mixture was centrifuged again for 5 min at 13,400 rpm. 100 μl of the supernatant were loaded onto a 96-well plate (Hartenstein, Würzburg, Germany). The absorbance of each mixture was measured at 610 nm using an Epoch spectrophotometer (BioTek, Bad Friedrichshall, Germany). To calculate the final absorbance of each single measurement, the mean absorbance of the blank control (1 M ascorbic acid) was subtracted from the absorbance of the relative mixture.

$$\begin{array}{l} \text{Absorbance}=\text{absorbance of the mixture}\\ -\text{absorbance of the blank control} \end{array}$$

### Odor-caffeine learning

Experiments were performed using standard methods (Niewalda et al., 2008; Schipanski et al., 2008; El-Keredy et al., 2012; Rohwedder et al., 2012; Apostolopoulou et al., 2013, 2014a). Petri dishes filled with a thin layer of 3.0% agarose were used containing either pure agarose or agarose plus caffeine at a concentration of 50 mM. As olfactory stimuli, we used 10 μl amyl acetate (AM; Fluka 46022; diluted 1:250 in paraffin oil, Fluka 76235) and benzaldehyde (BA; undiluted; Fluka 12010). Odorants were loaded into custom-made Teflon containers (4.5 mm diameter) with perforated lids. A first group of 30 animals was exposed to AM while crawling on agarose medium also containing caffeine as a negative reinforcer. After 5 min, larvae were transferred to a fresh, pure-agarose Petri dish and exposed to BA (AM+/BA). This cycle of training trials was repeated two more times. A second group of larvae received reciprocal training (AM/BA+). Then larvae were transferred onto test plates containing agarose plus caffeine on which AM and BA were presented on opposite sides. After 3 min, individuals were counted as located on the AM side (# AM), the BA side (# BA), or in a 10 mm neutral zone. We determined a preference index for each training group as follows:

$$\begin{array}{lll} \text{Pre}{\text{f}}_{\text{AM}+∕\text{BA}} & = & \left(\#\text{AM}-\#\text{ BA}\right)∕\#\text{ Total}\\ \text{Pre}{\text{f}}_{\text{AM}∕\text{BA}+}\; & = & \left(\#\text{AM}-\#\text{ BA}\right)∕\#\text{ Total} \end{array}$$

To measure specifically the effect of associative learning, we then calculated the associative performance index (PI) as the difference in preference between the reciprocally trained larvae:

$$\begin{array}{l} \text{PI }=\left(\text{Pre}{\text{f}}_{\text{AM}+∕\text{BA}}-\text{Pre}{\text{f}}_{\text{AM}∕\text{BA}+}\right)∕2 \end{array}$$

Negative PIs thus represent aversive associative learning. Division by two ensures scores are bound within [−1; 1]. The sequence of training trials (i.e., AM+/BA or BA/AM+) was alternated across repetitions of the experiment.

### Survival on caffeine diet

Experiments were performed using standard methods (Rohwedder et al., 2012; Apostolopoulou et al., 2014a). Vials prepared for control groups were filled with 1% (w/v) agarose solution. Vials prepared for experimental groups were filled with 1% (w/v) agarose plus 50 mM caffeine. Twelve wild-type first instar larvae were placed in each vial and kept at 25°C during the experiment. The number of surviving larvae was counted each day for 9 consecutive days. During the experiment, drops of tap water were occasionally added to the vials to prevent dehydration. The relative survival of the larvae, in each vial, was calculated every day by dividing the number of living larvae on this day with the total number of larvae on day 1.

$$\begin{array}{l} \text{Relative Survival}=\frac{\#\text{ living larvae on a specific day}}{\#\text{ total larvae on day }1} \end{array}$$

### Statistical methods

Kruskal–Wallis tests were performed and, in case of significance, followed by Wilcoxon rank-sum tests; Holm–Bonferroni corrections were used for multiple comparisons as applicable. Likewise, Wilcoxon signed-ranked tests were used to compare values against chance level. All statistical analyses were performed with R version 2.14.0 and Windows Excel 2010. Figure alignments were done with Adobe Photoshop. The behavioral data are presented as boxplots (middle line, median; box boundaries, 25%/75% quantiles; whiskers, 10%/90% quantiles; circles, outliers). Asterisks (^*^, ^**^, ^***^) and “n.s.” indicate *p* < 0.05, *p* < 0.01, *p* < 0.001, and *p* > 0.05, respectively.

### Scanning electron microscopy (SEM)

For SEM, larvae were bathed in hot water for 1.5–2 min. Fixation was carried out in 2.5% glutaraldehyde buffered in 0.05 M Na-cacodylate buffer (pH 7.4, 396 mOsm) at 4°C. After 30 min, approximately the anterior third was cut off and put back in fresh fixative for additional 18 h at 4°C. After fixation, samples were washed three times for 10 min, respectively, in 0.1 M Na-cacodylate (pH 7.4), followed by post-fixation in 1% osmium tetroxide (OsO_4_) for 2 h at 4°C. After additional washing steps (3 × 10 min with 0.2 M Na-cacodylate, pH 7.4), specimens were dehydrated in ascending ethanol concentrations and then transferred into a critical point device (Bal-Tec CPD 030, Liechtenstein) and dried via CO_2_. After mounting on aluminum stubs with CCC (Conductive Carbon Cement, Plano GmbH, Wetzlar, Germany), specimens were coated in a sputter coater (Balzers SCD 030, Liechtenstein) with 5 nm gold-palladium in order to enhance conductivity. Samples were examined in a FESEM Auriga TM Crossbeam workstation (Zeiss, Jena, Germany). Images were analyzed and processed with Image J software (http://imagej.nih.gov/ij).

### Light microscopy

Dissection of third instar larvae was performed in phosphate-buffered saline (PBS). After fixation in 3.7% formaldehyde (Merck, Darmstadt, Germany) in PBS for 30 min, heads were washed seven times in PBT (PBS with 3% Triton-X 100, Sigma-Aldrich, St. Louis, MO). Next, 5% normal goat serum (Vector Laboratories, Burlingame, CA) in PBT was added for 2 h. The primary antibody was applied for 2 days at 4°C. Samples were then washed six times with PBT. The secondary antibody was applied for 2 days at 4°C and specimens were washed eight times with PBT. Finally, samples were mounted in Vectashield (Vector Laboratories, Burlingame, CA) between two cover slips and stored at 4°C in the dark.

Anti-elav [Anti-elav mouse, DHSB (Iowa City, IA), 1:100] served to visualize neuronal nuclei in the periphery. As secondary antibody, IgG Alexa Fluor 647 (goat anti-mouse IgG Alexa Fluor 647 A21236; Molecular Probes, 1:200) was used. Images were obtained using a Zeiss LSM510 confocal microscope with a 25X oil immersion objective. Image stacks were projected and analyzed with Image J software (http://imagej.nih.gov/ij). Photoshop (Adobe Systems Inc., San José, CA) was used for contrast and brightness adjustment as well as for rotation and organization of the images.

### Calcium imaging

For calcium imaging experiments, third instar larvae that carried the genetically encoded calcium sensor *UAS-GCaMP6m* in the D1 (via *Gr93a-Gal4*) and D2 (via *Gr33a-Gal4*) pairs of neurons were used. Larvae were prepared as follows: First, two thirds of the caudal part of the larval body were removed to reduce body movements. Only the rostral part, including mouth hooks and the pharynx were kept. The head cuticle was opened dorsally to improve visibility of the GRNs. The preparation was fixed with minutien needles to allow visual access from dorsal direction to the DPS and was bathed in *Drosophila* saline solution (130 mM NaCl, 36 mM sucrose, 5 mM KCl, 5 mM HEPES, 2 mM CaCl_2_, and 2 mM MgCl_2_, pH 7.3). Movements of the mouth hooks and the anterior body part were not completely abolished in these preparations, but largely reduced. The calcium responses of the pair of D1 neurons to caffeine (25 mM), denatonium (10 mM), quinine (5 mM), theobromine (25 mM), canavinine (12.5 mM), salicin (12.5 mM), and fructose (25 mM) stimuli were recorded using a Zeiss Axio Examiner D1 microscope (Zeiss, Jena, Germany) equipped with a Zeiss water immersion objective (Zeiss W“PlanApochromat” DIC VIS-IR, 40X/1.0; Zeiss, Jena, Germany). Excitation light was provided by a 470 nm LED-Colibri Modul. The intensity of the excitation light was adjusted for every larva in the range between 5 and 10% to obtain similar basal fluorescence values. Emission light was recorded via the camera Axiocam 506 (Zeiss, Jena, Germany). Imaging frames were acquired with a frame rate of ~4 Hz.

### Gustatory stimulus application

Gustatory stimuli (dissolved in *Drosophila* saline) were applied with a custom-built gustatometer with computer-controlled valves into a flow chamber. The protocols for stimulus application started with a constant saline flow for 30 s to allow for recording of the background fluorescence. Then, gustatory stimuli were applied in a constant flow for 20 s, followed by 20 s bath application of the gustatory stimuli without flow. Finally, the preparation was washed with saline for 80 s before the recording was stopped.

### Data analysis

First, calcium imaging recordings were corrected for lateral movement artifacts. Using the function “Align slices in stack” in Image J software (http://imagej.nih.gov/ij). Afterwards, the calcium response time traces were extracted from the somata by selecting an elliptical region of interest (ROI). Movement in the z direction, when the neuron left the focal plane, was observed as strong fluorescence decreases in the recordings and in the resulting time traces. These z movement artifacts were not corrected and not visible in the final averaged values of several animals. Responses were calculated as the relative fluorescence change ΔF/F = (Fi − F0)/F0 [with Fi being the fluorescence value at each time point (i) during the recording, and F0 the mean of frames 60–99, before stimulus application]. Animals were pooled according to their genotype and type of gustatory stimulus. The mean time traces with the standard errors are shown.

The response delay (shown as seconds after stimulus onset) was calculated as the duration after stimulus onset, at which 10% of the maximum ΔF/F signal was reached. The response delay was calculated for each single animal. Then the animals were pooled according to their genotype to display the mean value and the standard error of the mean. Wilcoxon rank sum test was performed to test for significance between the genotypes.

## Results

### When exposed to caffeine *Drosophila* larvae initiate avoidance behavior, suppress feeding, establish aversive olfactory associations, and die earlier

#### Caffeine-dependent choice behavior

**Figure 1:**
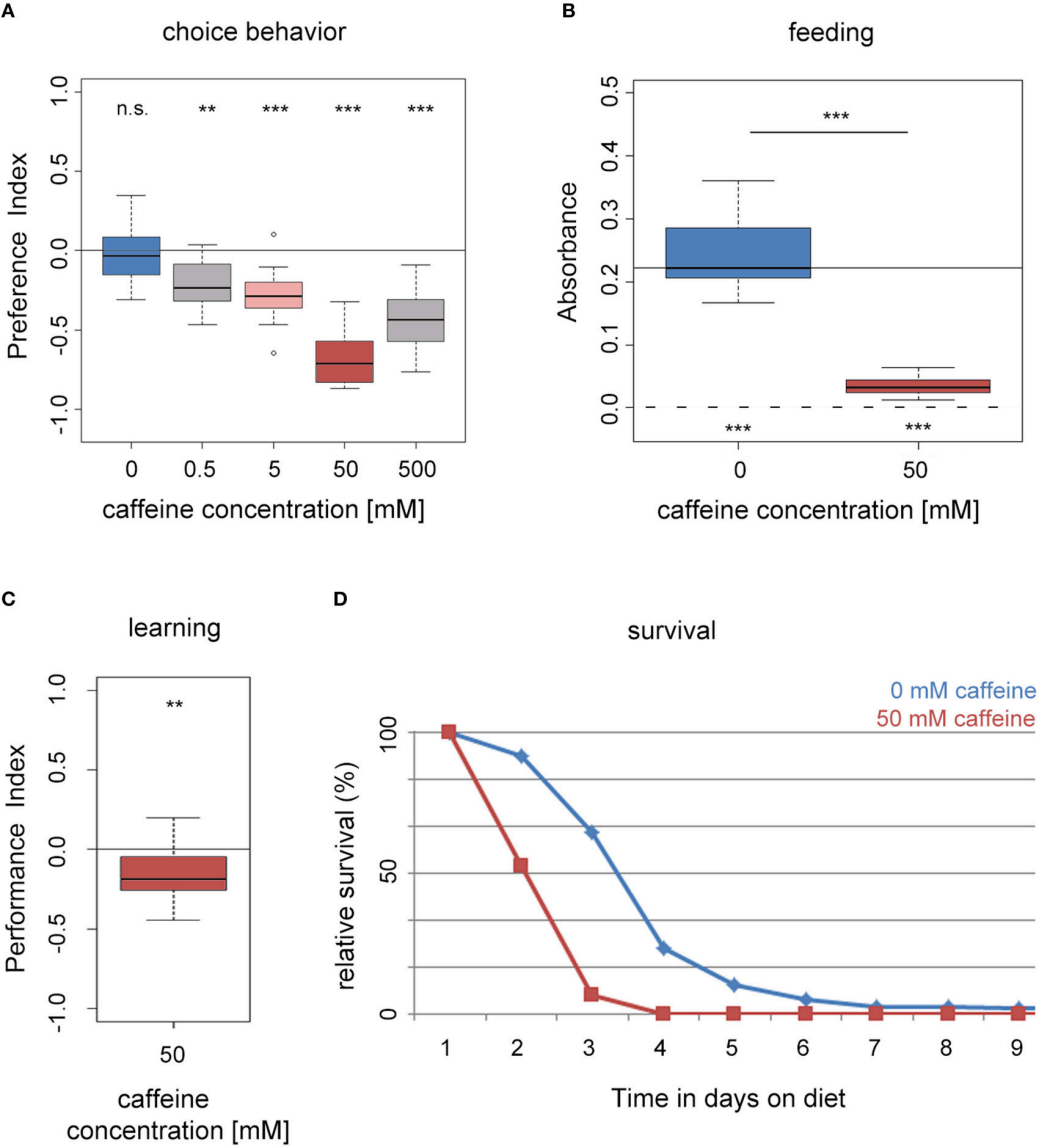
*****Drosophila*** larvae perceive caffeine as an aversive stimulus. (A)** Wild-type larvae avoid caffeine in concentrations ranging between 0.5 and 500 mM in a dose dependent manner (*n* = 15–16 for each concentration; *p* = 0.4953 for 0 mM, *p* = 0.0024 for 0.5 mM, *p* = 0.0006 for 5 mM, *p* = 0.0007 for 50 mM, and *p* = 0.0007 for 500 mM caffeine). **(B)** Feeding on a substrate that contains 50 mM caffeine (red box) is significantly reduced compared to baseline feeding on a pure agarose substrate (blue box; *n* = 15–16; *p* = 2 ^*^ 10^−6^). Yet, they did ingest low amounts of caffeine-containing food (when tested against zero *p* = 0.0005 and 0.0007 for 0 and 50 mM caffeine, respectively). The continuous line indicates the median absorbance at 0 mM caffeine. The dashed line indicates zero absorbance and thus no feeding. **(C)** Caffeine can act as a negative reinforcer in associative olfactory conditioning (*n* = 16; *p* = 0.0097). **(D)** Survival of wild-type larvae on an agarose substrate, which contains 50 mM caffeine (red) is reduced in comparison to survival on pure agarose substrate (blue; *n* = 15). Differences between groups are presented over the related box plots in **(B)**. Differences against a mean of 0 are shown above [in **(A)** and **(C)**] or below [in **(B)**] each box plot. n.s. non-significant *p* > 0.05, ^**^p < 0.01 or ^***^p < 0.001; small circles indicate outliers.

First, we assessed if naïve wild type larvae (WTCS) are attracted to caffeine or avoid it (Niewalda et al., 2008; Schipanski et al., 2008; El-Keredy et al., 2012; Rohwedder et al., 2012). On control Petri dishes, with only pure agarose, larvae distributed randomly (Figure 1A). When groups of larvae were placed on half–half test plates with one half containing pure agarose and the other half containing agarose with caffeine at concentrations ranging from 0 to 500 mM, larvae avoided the caffeine side. Increasing concentrations of caffeine resulted in increasing avoidance of the caffeine-containing medium with a maximum response at 50 mM (Figure 1A). Therefore, a concentration of 50 mM was chosen in most of the experiments. Please note that the avoidance at 500 mM is slightly reduced. A reason could be a harmful effect for high caffeine concentrations or the initiation of a more undirected escape response that was also seen for high agarose concentrations (Apostolopoulou et al., 2014b).

#### Feeding

We assessed whether larval feeding was altered on agarose containing 50 mM caffeine compared to a control medium of pure agarose only (Figure 1B). In these experiments, larvae did not have a choice of substrate, but they were allowed to eat different amounts of it (Niewalda et al., 2008; Schipanski et al., 2008; El-Keredy et al., 2012; Rohwedder et al., 2012). We evaluated the amount of consumed substrate by supplementing the food with dye (see Section Methods). Although larvae avoided caffeine when given a choice (Figure 1A), they did ingest caffeine-containing food in a non-choice situation. However, larvae consumed significantly less of the 50 mM caffeine-containing substrate compared to the control (Figure 1B).

#### Associative olfactory learning

After experiencing an odor together with high salt concentrations or quinine, *Drosophila* larvae learn to avoid that odor in a later test; hence, these gustatory stimuli can be used as negative reinforcers (unconditioned stimulus, US; Gerber and Hendel, 2006; Niewalda et al., 2008; Selcho et al., 2009; Schleyer et al., 2011; El-Keredy et al., 2012; Apostolopoulou et al., 2014a). Whether caffeine has a similar function for larvae was not tested to date. We trained larvae by presenting one odor with 50 mM caffeine and a second odor with pure agarose. In the subsequent test larvae could choose between the two odors in the presence of caffeine. Larvae avoided the odor previously paired with caffeine, indicating that under these circumstances 50 mM caffeine had negative reinforcing function (Figure 1C and Supplemental Figure 1B). In additional experiments, we found that the agarose concentration influenced the behavioral output (see also Apostolopoulou et al., 2014b). When the agarose concentration was reduced, the learning effect decreased (Supplemental Figure 1), indicating an interaction between caffeine and agarose concentration, possibly related to substrate stiffness.

#### Survival

We measured larval survival rates on caffeine by placing first instar larvae in vials that contained 50 mM caffeine mixed into agarose as their only food source (Figure 1D; Rohwedder et al., 2012; Apostolopoulou et al., 2014a). Compared to control animals in pure agarose vials, experimental larvae on 50 mM caffeine showed reduced survival. No survivors were left on caffeine after day 3, whereas on pure agarose survivors were still present after day 5 (Figure 1D).

In summary, our results show that larvae perceived caffeine as a negative stimulus. Larvae avoided and fed less on a caffeine containing substrate, they showed aversive olfactory learning, and they died earlier.

### Identification of *Gal4* driver lines that express in single gustatory neurons

**Figure 2:**
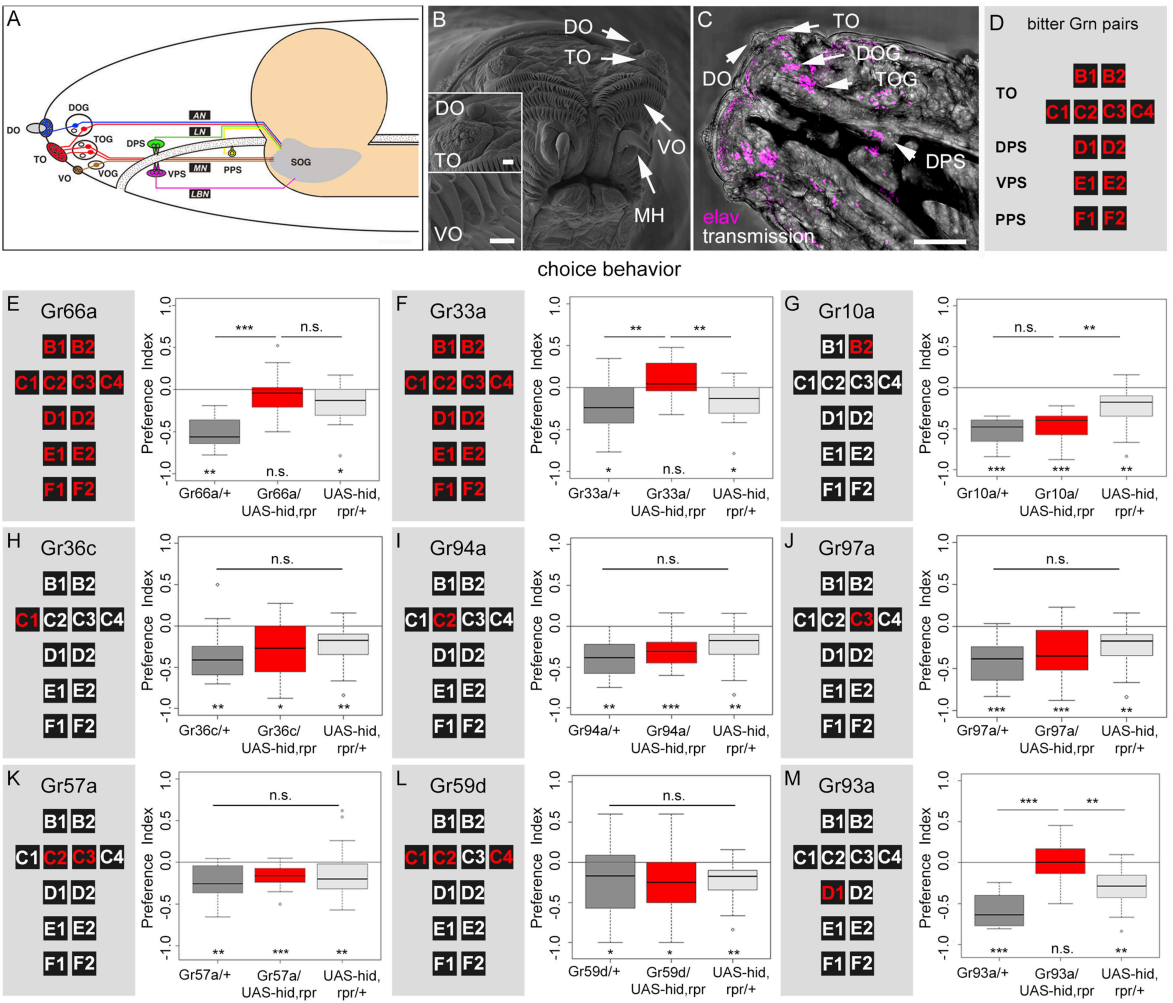
**A single pair of pharyngeal GRNs located in the DPS is necessary for caffeine dependent choice behavior. (A)** Schematic diagram of the larval gustatory system depicting the external (DO, dorsal organ; TO, terminal organ; VO, ventral organ) and internal gustatory organs (DPS, dorsal pharyngeal sense organ; VPS, ventral pharyngeal sense organ; PPS, posterior pharyngeal sense organ), their respective ganglia (DOG, TOG, VOG), and their connections (AN, antennal nerve, LN, labral nerve, MN, maxillary nerve, LBN, labial nerve) to the suboesophageal ganglion (SOG, modified from Python and Stocker, 2002). **(B)** High-resolution scanning electron microscope image of the larval head showing the DO, TO, VO, and mouth hooks (MH). Upper inset: detail view of DO and TO. Lower inset: detailed view of VO. The VO is located behind two rows of cirri. Scale bars: 20 μm, upper inset: 15 μm, lower inset: 2 μm, **(C)** Light microscopy: Dorsal view of the larval head region, which shows the DO, TO, and DPS. The ventral pharyngeal sensilla and the posterior pharyngeal sensilla are hidden under the pharynx and are not visible. DOG and TOG are visualized by marking all neurons using an anti-elav marker (magenta). Scale bar: 100 μm. **(D)** Schematic organization of the set of 12 bitter GRNs that is used to summarize the Gal4 line expression pattern in the following. **(E–M)** Screen to identify caffeine sensitive GRNs that instruct larval choice behavior. The left panels show a schematic representation of the 12 bitter neurons of the TO (B1, B2, C1–C4), DPS (D1 and D2), VPS (E1 and E2), and PPS (F1 and F2). The panels on the right show the results for caffeine-dependent choice behavior after ablation of the GRNs marked in red via the apoptosis inducing genes *hid* and *rpr*. Control genotypes are shown in gray, experimental groups at the center in red. Ablation of all 12 GRN pairs completely abolishes caffeine dependent choice behavior **(E,F**: *p* = 0.3635 and 0.1092, respectively), whereas in both cases genetic control groups avoid caffeine [in **(E)** *p* = 0.0011 for the Gal4 control and *p* = 0.0211 for the UAS-control; in **(F)** *p* = 0.0131 for the Gal4 control and *p* = 0.0211 for the UAS-control]. The same loss of caffeine dependent choice behavior is seen when specifically ablating the D1 GRN pair (*p* = 0.9499 against chance levels, *p* = 2 ^*^ 10^−5^ compared to the Gal4 control and *p* = 0.0051 compared to the UAS-*hid,rpr* control) **(M)**. No change in choice behavior was detected when ablating only B2 **(G)**, C1 **(H)**, C2 **(I)**, C3 **(J)**, C2 and C3 **(K)**, or C1, C2, and C4 **(L)** GRNs (*p* = 0.1406 when Gr10a/UAS-*hid,rpr* is compared to the Gal4 control and *p* = 0.0022 when compared to the UAS control; for all other experiments there was no difference between the three groups: *p* = 0.1349 for Gr36c, *p* = 0.0574 for Gr94a, *p* = 0.0504 for Gr97a, *p* = 0.1035 for Gr57a, and *p* = 0.2142 for Gr59d). Sample size for each group *n* ≥ 14; Significances against a mean of 0 are given at the bottom of each panel in **(E–M)**. Significances between experimental groups are depicted above the respective box plots in **(E–M)**. n.s. non-significant *p* > 0.05, ^*^p < 0.05, ^**^p < 0.01, and ^***^p < 0.001. Small circles indicate outliers. Please note that in **(E)** and **(F)** the same data is shown for the UAS control. The same is true for **(G–J)** and **(L)**.

How do *Drosophila* larvae perceive caffeine? Are there specific GRNs that respond to caffeine? First, we visualized the external sensory organs (Figure 2A) via scanning electron microscopy (SEM, Figure 2B). The DO at the tip of the larval head is a prominent multiporous cuticle structure and the principal olfactory organ. Sensilla surrounding the DO dome may serve gustatory and other sensory functions (Figure 2B; Singh and Singh, 1984; Python and Stocker, 2002; Klein et al., 2015; Ni et al., 2016). The TO is located in close proximity, ventral to the DO, and its sensilla respond to different modalities including gustation (Singh and Singh, 1984; Oppliger and Vlimant, 2000; Python and Stocker, 2002; Apostolopoulou et al., 2014a; Kim et al., 2016; van Giesen et al., 2016; Figure 2B). The VO is located on the ventral side of the cephalic lobes, covered by a row of cirri. It may also serve gustatory, as well as mechanosensory function (Figure 2B; Singh and Singh, 1984; Python and Stocker, 2002). Second, we visualized the individual somata located in the ganglion of the external and pharyngeal sensory organs by light microscopy using an anti-elav antibody (Figure 2C; Gendre et al., 2004). These organs served as a reference to study the neuronal expression patterns of different Gr-Gal4 lines in the entire set of GRN pairs (Figures 2A,D). Expression of UAS-*mCD8::GFP* (Ito et al., 1998) via the two driver lines *Gr66a-Gal4* and *Gr33a-Gal4* (Kwon et al., 2011; Weiss et al., 2011) confirmed that they were co-expressed in 12 GRN pairs. These 12 pairs were suggested to represent the entire set of larval bitter receptor neurons. They were: (i) two pairs B1 and B2 in the dorsolateral group of sensilla of the TO, (ii) four pairs C1–C4 in the distal group of TO sensilla, (iii) two pairs D1 and D2 in the DPS, (iv) two pairs E1 and E2 in the VPS, (v) two pairs F1 and F2 in the PPS (a detailed anatomical description is given in Figures 2A,D and Supplemental Figures 2A,B). Regarding single GRNs, we found that *Gr10a-Gal4* was expressed in B2 neurons, *Gr36c-Gal4* was expressed in C1 neurons, *Gr94a-Gal4* was expressed in C2 neurons, and *Gr97a-Gal4* was expressed in C3 neurons (Kwon et al., 2011; Apostolopoulou et al., 2014a; Supplemental Figures 2C–F). *Gr57a-Gal4* and *Gr59d-Gal4* labeled C2 and C3 neurons and C1, C2, and C4 neurons, respectively (Kwon et al., 2011; Apostolopoulou et al., 2014a). Both of these latter lines showed additional expression in the pharyngeal sensory system that was not further analyzed (Kwon et al., 2011). *Gr93a-Gal4* crossed with UAS-*mCD8::GFP* labeled the single D1 neuron pair (Supplemental Figure 2G) plus two olfactory receptor neurons (ORNs) in each DO, which projected to two glomeruli of the antennal lobe (Supplemental Figure 2H, see arrows). Please note that by using *Gr33a-Gal4* it was not possible to distinguish between individual GRNs in the same cluster (B1 and B2; C1–C4; D1 and D2; E1 and E2; F1 and F2). The identification of individual GRNs in *Gr10a-Gal4, Gr36c-Gal4, Gr94a-Gal4, Gr97a-Gal4, Gr57a-Gal4, Gr59d-Gal4*, and *Gr93a-Gal4* was verified based on the data of Kwon et al. (2011).

### TO neurons are not necessary for caffeine avoidance

To analyze if any of the above described GRNs were necessary for sensing caffeine, we crossed each Gal4 line with UAS-*hid,rpr*. Ectopic expression of *hid* and *rpr* induced apoptosis through caspase activation (White and Steller, 1995; White et al., 1996; Kurada and White, 1998). We tested experimental larvae and their appropriate controls for caffeine-dependent choice behavior. For both *Gr66a-Gal4* and *Gr33a-Gal4* we found—in contrast to the genetic controls—that ablating the complete set of 12 GRN pairs completely abolished the caffeine-dependent avoidance (Figures 2E,F). These results suggest that at least one of these 12 GRN pairs was necessary to induce caffeine avoidance behavior.

Ablation of B2 (Gr10a, Figure 2G), C1 (Gr36c, Figure 2H), C2 (Gr94a, Figure 2I), or C3 (Gr97a, Figure 2J) neurons at the TO did not alter larval caffeine avoidance. Ablation of multiple TO neuron pairs, like C2 and C3 (Gr57a, Figure 2K), or C1, C2, and C4 together (Gr59d, Figure 2L) also did not alter the caffeine avoidance of the larvae. We therefore conclude that these combinations of GRNs in the TO might not be necessary for caffeine-dependent choice behavior, although we cannot exclude that in individual animals always each GRN is ablated and that ablation of individual GRNs may lead to perturbing side effects at the entire TO.

### Caffeine avoidance is mediated by a single pair of D1 neurons in the DPS

To investigate the role of pharyngeal sensory neurons in caffeine perception we crossed *Gr93a-Gal4* with UAS-*hid,rpr* to induce apoptosis in the D1 neuron pair of the DPS (plus two ORN pairs). The resulting larvae did not display any caffeine avoidance behavior (Figure 2M) but where able to avoid quinine (Supplemental Figure 3), suggesting that caffeine sensing was abolished in this behavioral context.

Since *Gr93a-Gal4* additionally labeled two ORN pairs and was also found to be expressed in the third antennal segment of adult *Drosophila* (Menuz et al., 2014), we tested whether olfaction contributed to the behavioral response to caffeine. First, we tested whether caffeine could act as an olfactory rather than gustatory stimulus in this assay (Huser et al., 2012; Selcho et al., 2014; Rohwedder et al., 2016). Larvae were placed on an agarose test plate with a container that included 50 mM caffeine on one side and a second container that included no caffeine on the other side. The container prevented direct contact of gustatory organs with caffeine. As larvae distributed randomly on the test plate (Supplemental Figure 4A) we argued that larvae could not smell 50 mM caffeine. As a second control experiment, we used the *Orco* mutant, which shows normal gustatory behavior but fails to respond to a broad range of odors (Larsson et al., 2004). Consistent with previous results (Kim et al., 2016), *Orco* mutant larvae performed well in the caffeine-dependent choice assay (Supplemental Figure 4B). As in larvae all ORNs co-express *Orco*, we conclude that ORNs did not contribute to caffeine-dependent choice behavior. Hence, we conclude that the single *Gr93a-Gal4* positive gustatory neuron pair (D1) in the DPS was necessary to express caffeine avoidance (Figure 2M).

### D1 neurons respond to bitter substances including caffeine

**Figure 3:**
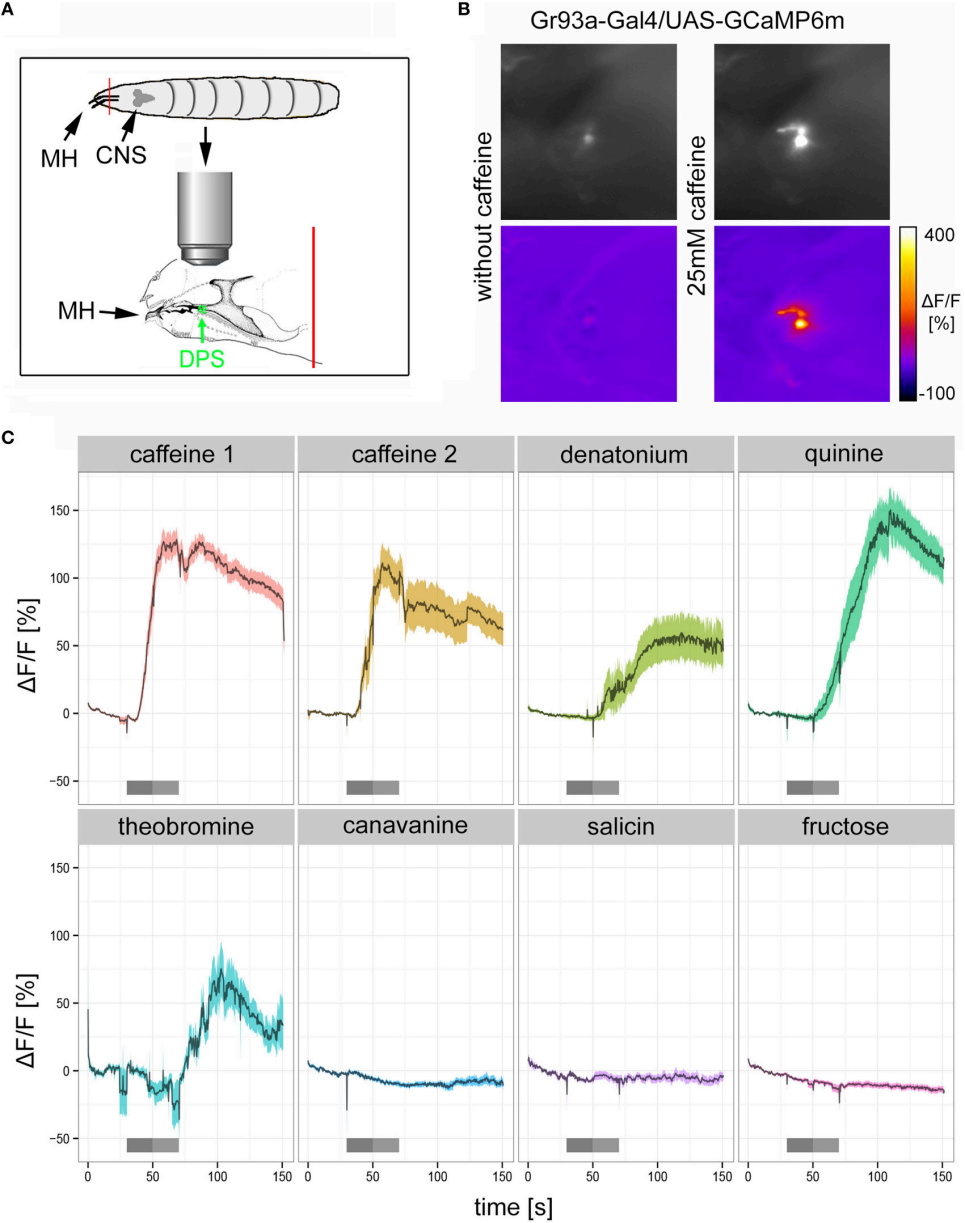
**The pharyngeal D1 GRN pair responds to caffeine stimulation. (A)** Schematic drawing of the preparation used for calcium imaging. The location of the DPS organ is highlighted in green. MH, mouth hooks; CNS, central nervous system. The red line indicates the place where the preparation was cut **(B)** Calcium increases of pharyngeal D1 neurons of *Gr93a-Gal4;*UAS-*GCaMP6m* larvae upon caffeine stimulation were recorded as fluorescence increases. The panel depicts the raw fluorescence images before and during caffeine application (25 mM) in a single larva preparation as morphological images (upper row), and the corresponding ΔF/F false color coded images (lower row). **(C)** Caffeine (25 mM) induced strong calcium responses of pharyngeal D1 neuron (red time trace), even when presented a second time to the preparation after extensive washing (yellow time trace). Denatonium (10 mM, light green trace), quinine (5 mM, dark green trace), and theobromine (25 mM, light blue trace) also induced calcium responses of the D1 neuron. In contrast, canavinine (12.5 mM, dark blue), salicin (12.5 mM, purple trace), and fructose (25 mM, magenta trace) did not elicit any responses. Responses are plotted as the relative response strength ΔF/F (*n* = 39, 12, 7, 11, 6, 7, 8, and 38, respectively; different animals were used in each group, individual animals were used for several stimuli). The dark gray bars below each trace indicate stimulus solution flow into the application chamber. The light gray bar indicates when the stimulus solution was present in the application chamber without flow. During all other time points a saline flow through the chamber washed out gustatory stimuli. Saline buffer did not trigger any neuronal responses.

If Gr93a neurons mediate caffeine aversion, they should respond to caffeine physiologically. To test this hypothesis, we expressed the calcium sensor GCaMP6m under the control of *Gr93a-Gal4* (Chen et al., 2013) and established a method to record intracellular calcium increases to bitter substance stimulation in cells of the DPS (Figure 3A). We found that 25 mM caffeine evoked strong intracellular calcium increases in the D1 neuron (Figures 3B,C: caffeine 1 shows the initial response of the cell to caffeine; caffeine 2 shows the response of the same cell to a second stimulation after an additional washing step). In addition, we detected calcium responses of the D1 neuron when stimulating with denatonium (10 mM), quinine (5 mM), and theobromine (25 mM). All of these responses were delayed as compared to the caffeine-dependent responses, suggesting some differences in sensitivity or the transduction mechanism. No calcium responses were detected with canavinine (12.5 mM), salicin (12.5 mM), or fructose (25 mM; Figure 3C). All substances except for fructose were reported to be bitter (König et al., 2014; Kim et al., 2016). In published behavioral studies, larvae only avoid denatonium, quinine, and canavinine, but do not respond to theobromine and salicin (König et al., 2014; Kim et al., 2016). Thus, denatonium and quinine avoidance may also be mediated by D1, while other neurons must be responsible for canavinine avoidance. It is puzzling, though, that D1 responded to theobromine, yet the animals did not avoid it (Kim et al., 2016). Thus, further work has to address how the physiological response of individual GRN pairs correlates with the avoidance behavior of the larva.

### D1 neurons are necessary for caffeine-induced learning

**Figure 4:**
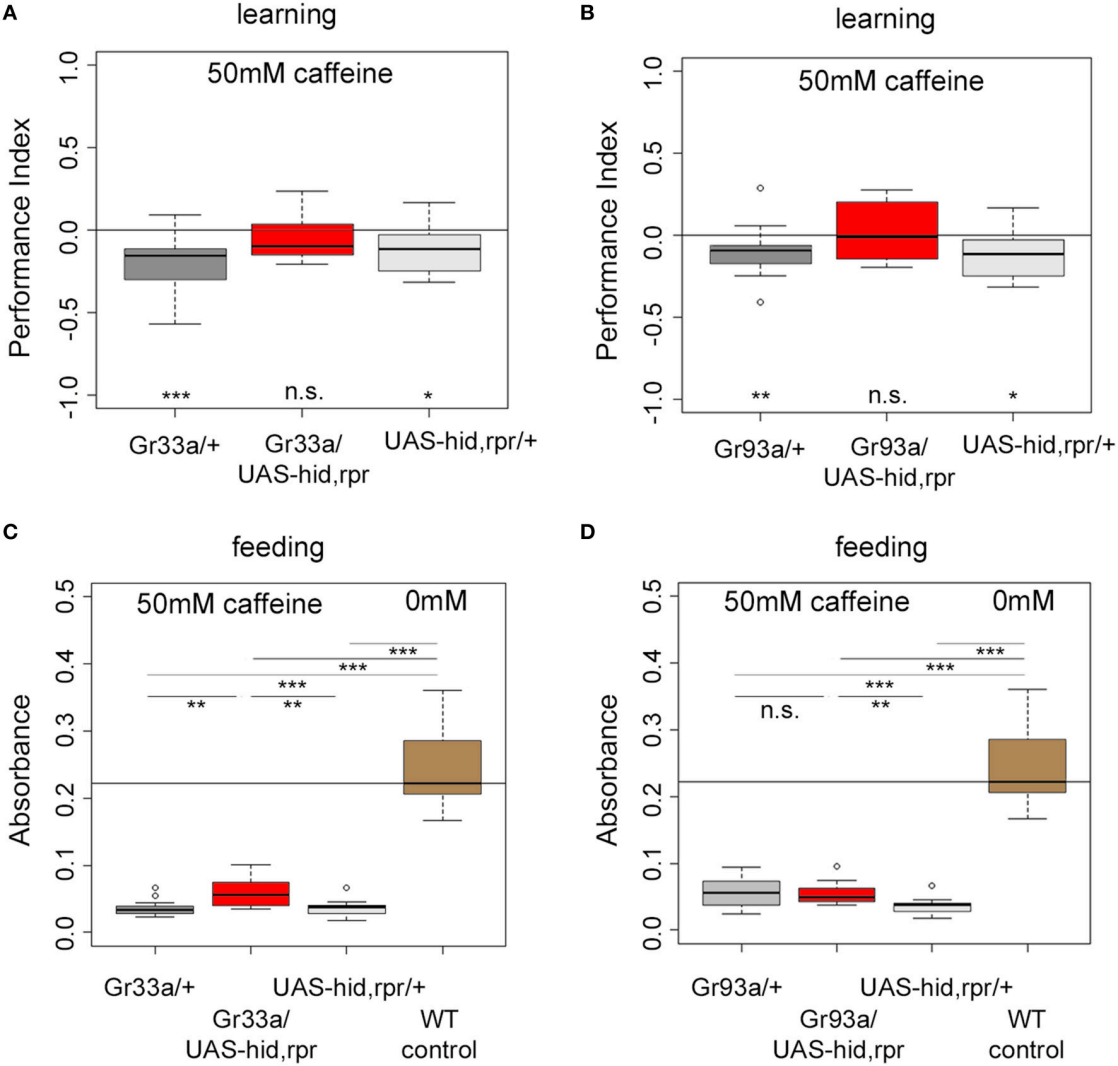
**The single pair of pharyngeal D1 GRNs is necessary for caffeine reinforced learning but dispensable for caffeine-dependent feeding. (A,B)** Associative olfactory learning: Genetic ablation of *Gr33a-Gal4*- or *Gr93a-Gal4*-positive GRNs via the apoptosis inducing genes *hid* and *rpr* completely abolishes 50 mM caffeine reinforced learning (for *Gr33a p* = 0.1591, for Gr93a *p* = 0.5282). Sample size for each group is *n* ≥ 15. **(C,D)** Feeding: Genetic ablation of *Gr33a-Gal4*- or *Gr93a-Gal4*-positive GRNs via the apoptosis inducing genes *hid* and *rpr* does not restore feeding on 50 mM caffeine substrate to baseline feeding levels on pure agarose substrate (*p* = 10^−5^ for *Gr33a*-Gal4/UAS-*hid,rpr* and *p* = 1.7750 ^*^ 10^−05^ for Gr93a-Gal4/UAS-*hid,rpr* compared to wild type controls), suggesting that these GRNs are not controlling feeding in this context. A slight but significant increase in feeding was detectable when ablating all twelve pairs of GRNs (*p* = 0.0011 compared to the Gal4 control, *p* = 0.0025 as compared to the UAS-*hid,rpr* control) **(C)**. This was not the case for the pharyngeal D1 GRN pair (*p* = 1.7750 ^*^ 10^−05^ compared to wild type controls, *p* = 0.9668 compared to the Gal4 control, and *p* = 0.0025 compared to the UAS-*hid,rpr* control) **(D)**. Sample size for each group *n* ≥ 12. Significances against a mean of 0 are given at the bottom of each panel in **(A)** and **(B)**. Significances between experimental groups are depicted above the respective box plots in **(C)** and **(D)**. n.s. non-significant *p* > 0.05, ^*^p < 0.05, ^**^p < 0.01, and ^***^p < 0.001. Small circles indicate outliers.

Do *Gr93a-Gal4* positive D1 neurons provide the sensory information about caffeine as a negative reinforcer? To answer this question, we ablated either the entire set of 12 pairs of bitter neurons using *Gr33a-Gal4*;UAS-*hid,rpr*, or the single pair of D1 neurons via *Gr93a-Gal4*;UAS-*hid,rpr*. Experimental larvae did not learn to avoid the caffeine-associated odor (Figures 4A,B). In contrast, Gal4 and UAS-*hid,rpr* control groups in both experiments were able to form odor-caffeine associations (Figures 4A,B). As this manipulation left task relevant odor-processing intact (Supplemental Figure 5), we conclude that D1 neurons were necessary to signal caffeine punishment in larvae.

### D1 neurons are not needed for caffeine dependent feeding

Are D1 neurons also needed to sense caffeine in other behavioral contexts, specifically when feeding? *Drosophila* larvae ate low amounts of caffeine-containing food when they do not have another choice (Figure 1B). This allowed us to ask whether *Gr93a-Gal4* D1 neurons were also necessary for caffeine driven feeding suppression, or whether there may be an additional caffeine-sensing channel required for feeding.

Ablating the 12 pairs of *Gr33a-Gal4* neurons only marginally increased feeding on a 50 mM caffeine-containing substrate when compared to both genetic controls (Figure 4C). The amount of consumed caffeine-containing substrate was clearly reduced compared to baseline feeding on caffeine-free agarose substrate (Figure 4C). Next, we tested feeding behavior on caffeine-containing substrates of larvae ablated of D1 neurons. There was no explicit effect on feeding (Figure 4D). The performance of larvae without D1 neurons was similar to the Gal4 control larvae but different from UAS-*hid,rpr* control larvae. The amount of consumed caffeine-containing substrate was clearly reduced compared to baseline feeding on caffeine-free agarose substrate, too. Our data suggest that the D1 neuron pair was not required for caffeine-dependent feeding avoidance, unlike as observed in case of caffeine-dependent choice behavior and caffeine-dependent learning. Other neurons in the *Gr33a* group partially contributed to caffeine feeding avoidance, even though they were not involved in choice behavior. Furthermore, since this effect was not complete, it suggests that other, so far unidentified, mechanisms contribute to caffeine driven feeding suppression.

### The caffeine receptor is likely a heteromultimer

**Figure 5:**
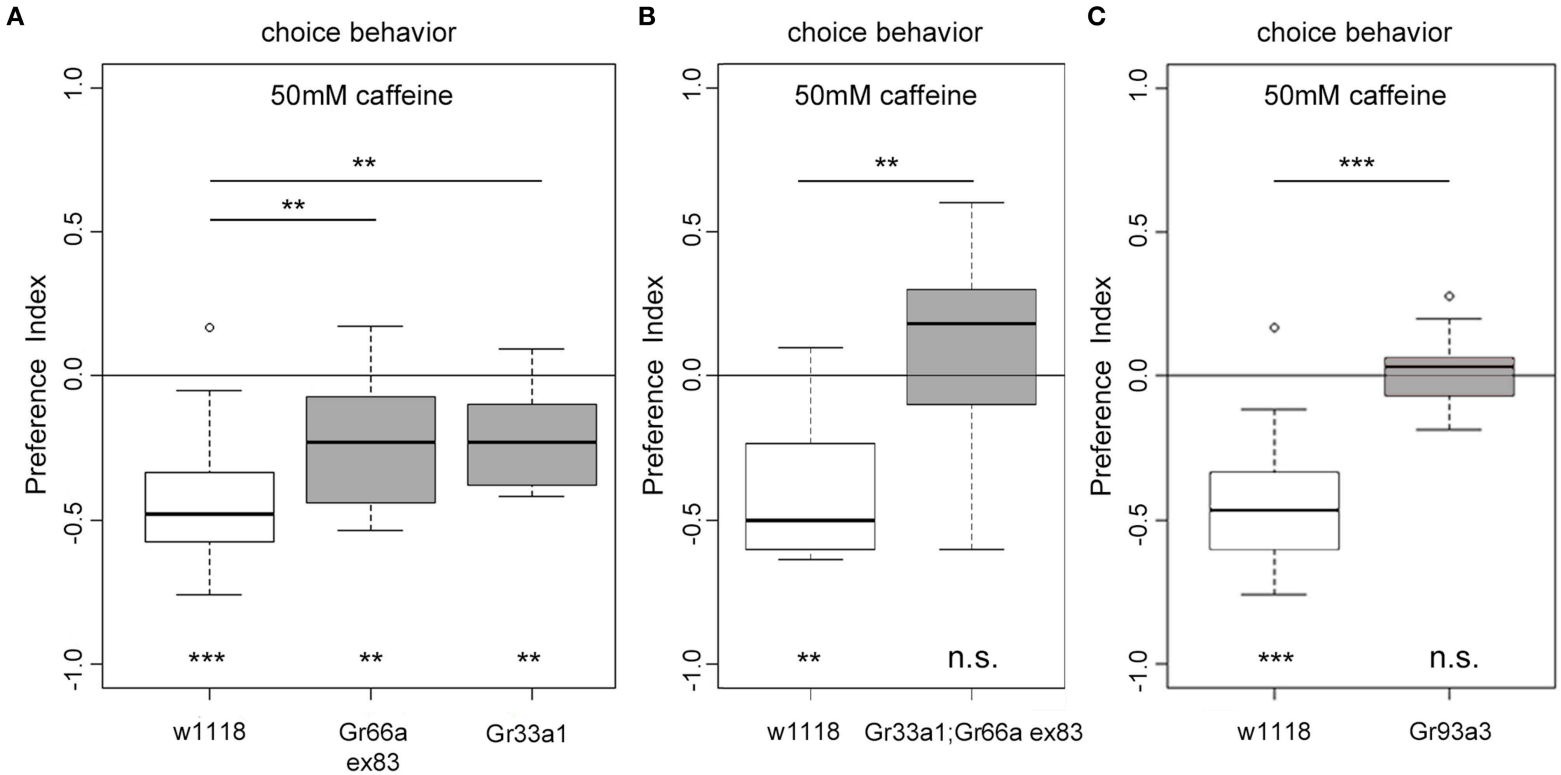
**The receptor genes ***Gr66a***, ***Gr33a***, and ***Gr93a*** are necessary for caffeine-dependent choice behavior. (A)** *Gr66a* and *Gr33a* receptor gene mutants (*Gr66aex83* and *Gr33a1*, respectively) show reduced caffeine-dependent choice behavior compared to the *w1118* control larvae (*p* = 0.0061 for *Gr66ex83* and *p* = 0.0044 for *Gr33a1*) but can still avoid caffeine (*p* = 0.0061 for *Gr66ex83* and *p* = 0.0021 for *Gr33a1* against chance levels). **(B)** *Gr33a1;Gr66aex83* double mutants do not show any caffeine-dependent choice behavior (*p* = 0.4124 against chance levels and *p* = 0.0046 for *Gr33a1;Gr66aex83* compared to *w1118*). **(C)** Gr93a receptor gene mutant larvae (Gr93a3) show no caffeine-dependent choice behavior (*p* = 0.7474). Sample size for each group is *n* ≥ 11 experiments, with 30 larvae each. Significances against a mean of 0 are given at the bottom of each panel. Differences between experimental groups are depicted above the respective box plots; n.s. non-significant *p* > 0.05, ^**^ *p* < 0.01, and ^***^ *p* < 0.001. Small circles indicate outliers.

Gr93a neurons co-express at least *Gr66a* and *Gr33a* receptor genes (Kwon et al., 2011). But how do these receptors contribute to caffeine perception? Adult flies lacking one of the *Gr66a, Gr33a*, or *Gr93a* receptor genes show impaired caffeine avoidance and no electrophysiological responses to caffeine (Moon et al., 2006, 2009). Thus, we assessed *Gr66aex83* (Moon et al., 2006) and *Gr33a1* (Moon et al., 2009) receptor gene mutants in the 50 mM caffeine-dependent choice assay. Both mutants showed reduced caffeine avoidance as compared to the *w1118* control larvae (Figure 5A). However, they showed significant residual caffeine-dependent choice behavior different from random distribution (Figure 5A). Larvae carrying the double mutation *Gr33a1; Gr66aex83* for both receptor genes were completely unable to avoid caffeine and accordingly behaved differently from control larvae (Figure 5B). In addition, we tested *Gr93a3* receptor gene mutant larvae (Lee et al., 2009), which failed to avoid caffeine (Figure 5C). Therefore, Gr93a, *Gr66a*, and *Gr33a* were necessary for caffeine sensing. *Gr66a* and *Gr33a*, however, could partially compensate for each other's loss.

**Figure 6:**
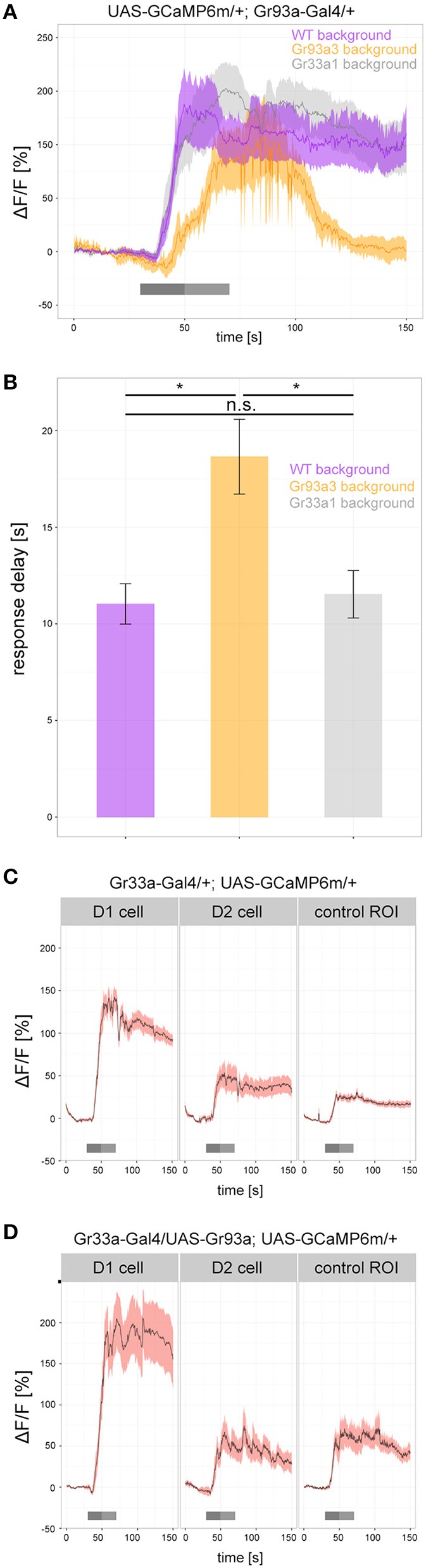
*****Gr93a*** receptor gene expression in the pharyngeal D1 neuron is necessary to elicit proper caffeine-dependent responses. (A)** Response profiles of the pharyngeal D1 neuron upon 25 mM caffeine stimulation in wild-type larvae (purple), *Gr93a3* mutant background larvae (orange), and *Gr33a1* mutant background larvae (gray). To this end, we introduced *Gr93a-Gal4; UAS-GCaMP6m* into three different genetic backgrounds. *Gr93a3* mutants showed a delayed response to caffeine that quickly disappeared in comparison to wild type and *Gr33a1* mutant larvae. Responses were plotted as the relative response strength ΔF/F (*n* = 8, 6, 8, respectively). Gray bars indicate stimulation as in Figure 3. (B) Detailed evaluation of the response delay in seconds of the three measurements shown in (A). The response delay was calculated as the time it takes from stimulus onset, until 10% of the maximum ΔF/F response is reached. Barplots show the mean response delay of the same animals as in (A). Differences between experimental groups are depicted above the respective box plots; n.s. non-significant *p* > 0.05, ^*^p < 0.05 tested with Wilcoxon rank sum test. (C) In contrast to the pharyngeal D1 neuron, no response to 25 mM caffeine was seen for the pharyngeal D2 neuron in *Gr33a-Gal4*; UAS-*GCaMP6m* larvae. The increase in fluorescent light observed in D2 is similar to that in a control background region in similar proximity to D1. Number of analyzed neurons: *n* = 18, 12, and 10, respectively. (D) Ectopic expression of *Gr93a* receptor gene in D2, is not sufficient to elicit a caffeine-dependent response. D1 neurons were activated by caffeine, but D2 neurons showed a similar response to a control region in *Gr33a-Gal4;* UAS-*Gr93a;* UAS-*GCaMP6m* larvae. Number of analyzed neurons: *n* = 16, 6, and 5, respectively.

To determine how Gr93a, *Gr66a*, and *Gr33a* contributed to the physiological response of the D1 neuron, we analyzed caffeine dependent calcium responses (25 mM) of the D1 neuron in a wild type background, in a *Gr93a3* homozygous mutant background and in a homozygous *Gr33a1* mutant background (Figure 6). All groups showed strong responses after caffeine application, but their time traces differed: without a functioning *Gr93a* gene, calcium response onset was delayed, and returned to baseline quicker (Figures 6A,B). Thus, *Gr93a* gene function was necessary for a proper physiological response of the D1 neuron.

Having observed that Gr93a was necessary, we next asked whether this gene was sufficient to provide a GRN with the function to respond to caffeine. We expressed UAS-*GCaMP6m* via *Gr33a-Gal4*. *Gr33a-Gal4* drove calcium sensor expression in the D1 neuron pair of the DPS that responded to caffeine (Figures 3B,C, 6C) but also in the D2 neuron pair that showed no caffeine response (Figure 6C and Supplemental Figure 6). The fluorescent increase found in D2 was entirely due to scattered light from the D1 cell, as shown by comparison with an unlabeled tissue area. D2 neurons were reported to co express the *Gr33a* and *Gr66a* genes but not the *Gr93a* gene (Kwon et al., 2011). We then used *Gr33a-Gal4* to drive both UAS-*GCaMP6m* and UAS-*Gr93a*, thus overexpressing the *Gr93a* receptor gene in D1 neurons and artificially expressing it in D2 neurons. However, while D1 gave increased responses as compared to a wild type background, suggesting that *Gr33a-Gal4* was strong enough to drive expression of Gr93a to physiological effects, D2 did not show any caffeine-dependent calcium increase (Figures 6C,D). We conclude that *Gr93a* gene function was not sufficient to provide a GRN with the ability to physiologically respond to caffeine, even in a *Gr33a* and *Gr66a* positive background, suggesting that additional components would be necessary for a functional caffeine receptor protein complex.

## Discussion

### Pharyngeal taste processing in larvae

Analysis of bitter taste processing in *Drosophila* larvae, similar to its adult counterpart, focuses almost exclusively on the external sensory system [(Kwon et al., 2011; Mishra et al., 2013; Alves et al., 2014; Apostolopoulou et al., 2014a; König et al., 2014; Kim et al., 2016; van Giesen et al., 2016) for the adult pharyngeal system see LeDue et al., 2015]. Although there is growing interest in mechanisms following food ingestion, the pharyngeal sense organs remain basically unexplored (Hergarden et al., 2012; Manzo et al., 2012; Marella et al., 2012; Pool and Scott, 2014; Pool et al., 2014; LeDue et al., 2015; Yapici et al., 2016).

Here, we show—to our knowledge—for the first time a taste dependent function for the larval pharyngeal sense organs. Larvae perceive their environment with a small number of GRNs that are occasionally genetically accessible on the single cell level (Kwon et al., 2011; Apostolopoulou et al., 2014a). Until now several studies identified single GRNs located at the external TO that are necessary for particular aspects of bitter food avoidance. C7 neuron pair function is required to keep larvae away from quinine and denatonium (van Giesen et al., 2016). C3 neuron pair function is necessary for larvae to avoid quinine and its activation suffices to trigger avoidance (Apostolopoulou et al., 2014a). Yet, although certain aspects of bitter sensation can be attributed to individual GRNs, it is likely that some bitter chemicals are not only perceived by single neuron pairs of the TO, but rather by an ensemble of gustatory neurons. GRN inactivation by Gr-Gal4 drivers such as *Gr66a-Gal4, Gr33a-Gal4, Gr59d-Gal4, Gr97a-Gal4, Gr57a-Gal4, Gr9a-Gal4, Gr23a-Gal4*, or *GMR57B04-Gal4* reduces or even inverts larval avoidance of quinine (Apostolopoulou et al., 2015; Kim et al., 2016; van Giesen et al., 2016). These results suggest that the C1, C2, C4 neuron pairs and the pharyngeal system are also required for quinine perception, in addition to C3 and C7 function (Apostolopoulou et al., 2014a; Kim et al., 2016; van Giesen et al., 2016). Yet, the precise mechanism of cooperation remains unclear. Partially because the analysis of larval taste is currently limited to only about one third (12 of the total 37) of the GRNs of the TO due to as yet missing genetic tools (Apostolopoulou et al., 2015). In addition, taste coding in larvae likely includes multiple levels of interaction: the sensory level, the subesophageal ganglion (SOG, the first taste integration center of the brain) and subsequent circuits in higher brain and premotor areas.

**Figure 7:**
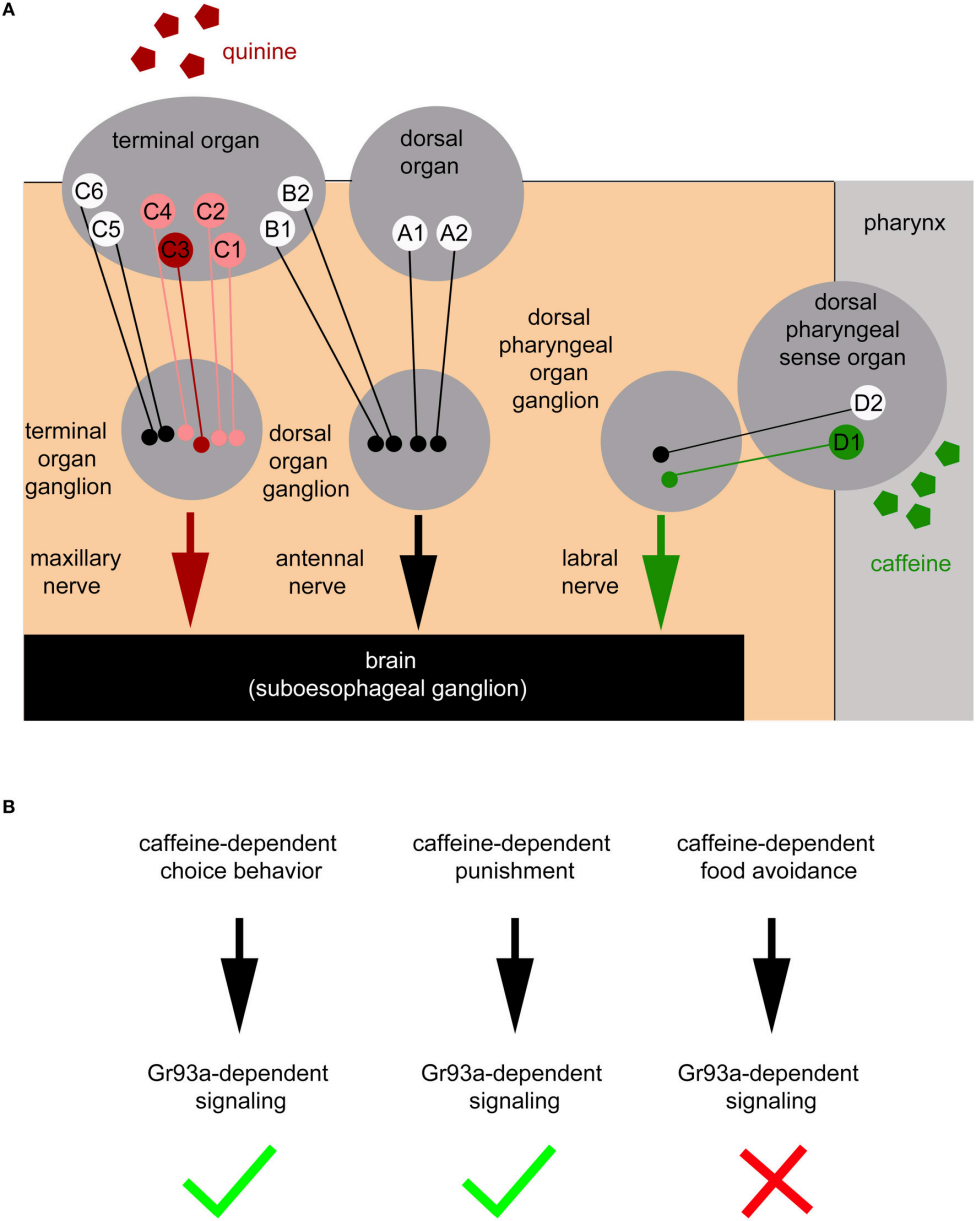
**Schematic overview of the larval peripheral sensory system perceiving caffeine and quinine taste to trigger larval behavior**. **(A)** Bitter quinine taste information affecting larval choice behavior is mediated mainly by the TO neurons C1–C4 (light red and red) and especially by the single TO neuron C3 (red). Bitter caffeine taste information affecting larval choice behavior is mediated by the single D1 neuron (green) of the DPS (this study). Ablation of D1 does not impair quinine-dependent choice behavior (Supplemental Figure 3). The ligand specificity for the rest of the 12 “bitter” neurons of the TO (B1, B2, C5, and C6), DO (A1 and A2), DPS, VPS, and PPS is yet unknown (indicated in white). Signals propagate to the subesophageal ganglion via the maxillary nerve (for quinine) and the labral nerve (for caffeine). From here, postsynaptic, yet unidentified, second order neurons further process gustatory signals to trigger taste-dependent choice behavior. In addition to the peripheral sensory system depicted here, this study suggests that there are more central sensors, which remain unidentified and are, therefore, not included in the scheme. **(B)** Taste information of the Gr93a positive D1 sensory neuron of the DPS is able to instruct caffeine-dependent behaviors. This includes caffeine-dependent choice behavior and caffeine-dependent punishment (necessary for aversive olfactory learning), but excludes a necessity for caffeine-dependent food avoidance.

Here, we show that the pharyngeal D1 neuron pair of the DPS constitutes an additional sensory organ level for bitter sensing (Figures 7A,B). Larvae without the D1 neuron pair do not show caffeine avoidance (Figure 2M) and odor-caffeine learning (Figure 4B). The D1 neuron pair responds to caffeine (Figure 3C). Sugar may be similarly sensed in the pharyngeal organ: adult *poxn* mutant flies lacking all external GRNs are still capable of selecting sugar by virtue of pharyngeal taste neurons (LeDue et al., 2015), while larvae lacking *Gr43a*, their main sugar receptor, fail to prefer sugar. Gr43a is not expressed in the larval external taste organs: the DO, TO, and VO. But, among other cells, the receptor is present in pharyngeal sensory neurons (Mishra et al., 2013). Therefore, the larval pharyngeal sensory organs appear to perceive tastes with opposing valence and to contribute to taste guided behaviors.

Yet, it was also shown that taste coding is more combinatorial than initially thought. Bitter tastants can suppress the stimulatory effect of attractive gustatory cues. This could take place in the taste receptor cells or in higher-processing central pathways (shown for different insects in Haskell and Schoonhoven, 1969; Dethier, 1978; Chapman et al., 1991; Simpson et al., 1991; Meunier et al., 2003; Gordon and Scott, 2009; Yong Jeong et al., 2013; Chu et al., 2014). Thus, interference with pharyngeal D1 neuron pair function could shift the net output of the entire taste system toward a more positive value that would also lead to an impairment in caffeine driven behaviors.

Interestingly the D1 neuron pair of the DPS is among the few larval sensory neurons to survive remodeling during metamorphosis as it is incorporated into the adult pharyngeal sensory system (Gendre et al., 2004). Tracing the D1 neuron pair throughout development would allow for analyzing its significance for adult taste perception. Further studies will be necessary in adult and larval *Drosophila* in order to dissect the detailed nature of taste coding via the external and pharyngeal taste organs, and its persistence across metamorphosis.

### Caffeine perception and feeding behavior

All growth in *Drosophila* normally occurs during the juvenile larval stages, resulting in a remarkable ~200-fold increase in body mass (Church and Robertson, 1966). To reliably recognize nutrients [e.g., yeast, the major source of proteins (Cooper, 1960), or carbohydrates (Mishra et al., 2013; Schwarz et al., 2014)] and to weigh them against low concentrations of bitter, potentially hazardous compounds, larvae need a sophisticated sense of taste.

We speculate that in contrast to food choice, consumption of bitter food in non-choice situations is only marginally influenced by TO and pharyngeal sensory organ function. *Drosophila* larvae consume less food if it contains caffeine or quinine (Figure 1; Apostolopoulou et al., 2014a). Ablation of the entire set of external and pharyngeal bitter GRNs via *Gr33a-Gal4* only marginally increases the amount of consumed caffeine or quinine containing substrate (Figure 4; Apostolopoulou et al., 2014a). We therefore argue that larvae have at least one additional system that perceives bitter information to regulate food consumption. This could be another, putative, caffeine sensitive receptor, or an allosteric effect of caffeine onto a sugar receptor or other receptor with positive valence. Alternatively, or additionally, caffeine may act via mechanisms that change the motivation or the health of the animals after consuming caffeine. Feeding in most insects can be divided into at least four phases: food finding, sampling, food consumption, and cessation of feeding (Beck, 1965; Schoonhoven, 1972). The decision as to whether and when a food source is accepted occurs during the first two phases. Our results suggest that the decision to avoid food that contains bitter substance like quinine, denatonium, or caffeine is made by the TO and/or the pharyngeal DPS. Once the decision is made to constantly feed, larvae start a motor program of alternating biting and non-biting periods (Ma, 1972; Chapman, 1982). Our data suggest that food consumption is under control of a different, yet unknown, system, because larvae that lack the 12 pairs of bitter GRNs still fed less on a caffeine containing substrate (Figure 4C). This would allow larvae to appropriately adjust their feeding rates in an environment that does not allow for food finding and sampling, e.g., due to the omnipresence of a harmful substance in the food source, as in our non-choice feeding assay.

Which systems may instruct food consumption? One possibility is the enteric system, which includes neurons in the esophageal, hypocerebral, and proventricular ganglion. In insects the enteric system regulates rhythmic foregut and pharynx movements and processes associated with these movements, like food consumption (Hill et al., 1966; Griss et al., 1991), air swallowing (Carlson and O'gara, 1983), and molting-related behaviors (Miles and Booker, 1998; Bestman and Booker, 2003). Further downstream along the digestive tract, enteroendocrine cells located in the midgut of adult *Drosophila* express Grs necessary for bitter taste perception. Enteroendocrine cells, in insects as well as in mammals, produce regulatory peptides upon detection of luminal nutrients or chemicals to regulate gut physiology, food intake, and glucose homeostasis in a paracrine and/or endocrine manner. By testing Gr93a mutants in the non-choice feeding assay it is possible to test this hypothesis. A reduction in feeding would support our interpretation, whereas normal feeding rates would argue for one or more Gr independent mechanisms.

### The molecular basis of caffeine perception

Caffeine, a methylxanthine primarily derived from coffee trees and tea plants, but also present at low concentration in fruits, is one of the behaviorally active substances most commonly consumed by humans (Clifford, 1985; Wintgens, 2012). Caffeine is known to improve alertness and arousal in humans and other mammals but also in invertebrates (Clifford, 1985; Wintgens, 2012). It has an impact on numerous insect behaviors, including fine motor movements, attention, and complex cognitive processes (Mustard, 2014). However, unlike most tastants, which are detected through G protein-coupled receptors at the cell surface, it has been proposed to also serve conserved pharmacological functions; namely, to increase cAMP through the inhibition of phosphodiesterases, to increase intracellular calcium levels via release of intracellular stores through ryanodine receptors, and as an antagonist of adenosine receptors (reviewed in Mustard, 2014).

Yet, in adult *Drosophila Gr33a* and *Gr66a* gene function—together with *Gr93a*—was demonstrated to be required for caffeine responsiveness (Moon et al., 2006, 2009; Lee et al., 2009). *Gr33a* was suggested to be a receptor required for the perception of many different bitter substances and was, therefore, proposed to function as a co-receptor for bitter sense in general (Moon et al., 2009). *Gr66a* and Gr93a, however, show a specific response to caffeine and other methylxanthine derivatives such as theophylline (Lee et al., 2009). In line with these results Gr93a mutant larvae show an altered physiological response of the D1 neuron pair to caffeine (Figures 6A,B) and *Gr33a*; *Gr66a* double mutant and Gr93a mutant larvae do not avoid caffeine (Figures 5B,C). Accordingly, caffeine sensing in *Drosophila* at different developmental stages functions through a specific (and more sensitive) Gr-dependent molecular mechanism, rather than an unspecific effect on internal calcium stores. However, the neuronal Gr93a expression differs between the larval and adult stage. Adult *Drosophila*—in contrast to larvae—show Gr93a expression in GRNs located at the periphery in different labellar sensilla (S_0_, S_1_, S_2_, S_6_, S_7_, and S_10_; Weiss et al., 2011) and the abdomen (Kwon et al., 2014). Therefore, it is likely that caffeine is differently perceived by larval and adult systems. Yet, a similar approach in adults would require intersectional techniques (LeDue et al., 2015) to clearly disentangle the pharyngeal system from the peripheral ones.

In mammals, taste receptors are either homo- or heterodimers, while olfactory receptors are homomeric proteins. *Drosophila* olfactory receptors (ORs) appear to be heterodimers comprised of *Orco* in combination with one additional OR (Larsson et al., 2004). The *Drosophila* CO_2_ receptor is a heterodimer consisting of Gr21a and Gr63a (Kwon et al., 2007). Here we show that caffeine perception via the pharyngeal D1 neuron requires at least four subunits, since misexpression of Gr93a, *Gr33a, Gr66a* in the pharyngeal D2 neuron is not sufficient to confer caffeine sensitivity to this neuron. Alternatively, the D2 neuron might be missing an element of the transduction cascade needed in D1, even though D2 is also a taste receptor neuron. Based on the recent findings of Delventhal and Carlson (2016) it is also possible that bitter signaling in larvae follows a more complex logic of interaction for Gr genes that also includes inhibiting effects. This organization seems to be conserved throughout *Drosophila* development as in adult flies misexpression of the three Grs does not equip a sugar responsive GRNs with caffeine sensitivity (Lee et al., 2009). Clearly, taste perception in *Drosophila*—and in particular in the *Drosophila* larva—has many mysteries yet to be solved.

## Author contributions

AA designed and performed the experiments, analyzed the data, and wrote the manuscript. SK, BS, ML, AW, and LM performed the experiments and analyzed the data. AR, CG, AL, and AT designed the experiments, analyzed the data, and wrote the manuscript.

## Funding

The work was supported by the DFG grants (TH1584/1-1) and (TH1584/3-1), the Baden-Württemberg Stiftung (all to AT), and the Zukunftskolleg of the University of Konstanz (to CG and AT).

### Conflict of interest statement

The authors declare that the research was conducted in the absence of any commercial or financial relationships that could be construed as a potential conflict of interest.

## References

[B1] Abbott J. (2014). Self-medication in insects: current evidence and future perspectives. Ecol. Entomol. 39, 273–280. 10.1111/een.12110

[B2] Alves G. Salle J. Chaudy S. Dupas S. Maniere G. (2014). High-NaCl perception in *Drosophila melanogaster*. J. Neurosci. 34, 10884–10891. 10.1523/JNEUROSCI.4795-13.2014 25122890 PMC6705259

[B3] Apostolopoulou A. A. Hersperger F. Mazija L. Widmann A. Wust A. Thum A. S. (2014b). Composition of agarose substrate affects behavioral output of Drosophila larvae. Front. Behav. Neurosci. 8:11. 10.3389/fnbeh.2014.00011 24478658 PMC3904111

[B4] Apostolopoulou A. A. Mazija L. Wust A. Thum A. S. (2014a). The neuronal and molecular basis of quinine-dependent bitter taste signaling in Drosophila larvae. Front. Behav. Neurosci. 8:6. 10.3389/fnbeh.2014.00006 24478653 PMC3902218

[B5] Apostolopoulou A. A. Rist A. Thum A. S. (2015). Taste processing in Drosophila larvae. Front. Integr. Neurosci. 9:50. 10.3389/fnint.2015.00050 26528147 PMC4602287

[B6] Apostolopoulou A. A. Widmann A. Rohwedder A. Pfitzenmaier J. E. Thum A. S. (2013). Appetitive associative olfactory learning in Drosophila larvae. J. Vis. Exp. e4334. 10.3791/4334 23438816 PMC3601210

[B7] Barretto R. P. Gillis-Smith S. Chandrashekar J. Yarmolinsky D. A. Schnitzer M. J. Ryba N. J.. (2015). The neural representation of taste quality at the periphery. Nature 517, 373–376. 10.1038/nature13873 25383521 PMC4297533

[B8] Beck S. D. (1965). Resistance of plants to insects. Annu. Rev. Entomol. 10, 207–232. 10.1146/annurev.en.10.010165.001231

[B9] Bernays E. A. Singer M. S. (2005). Insect defences: taste alteration and endoparasites. Nature 436, 476. 10.1038/436476a 16049466

[B10] Bestman J. E. Booker R. (2003). Modulation of foregut synaptic activity controls resorption of molting fluid during larval molts of the moth *Manduca sexta*. J. Exp. Biol. 206, 1207–1220. 10.1242/jeb.00237 12604581

[B11] Bray S. Amrein H. (2003). A putative Drosophila pheromone receptor expressed in male-specific taste neurons is required for efficient courtship. Neuron 39, 1019–1029. 10.1016/S0896-6273(03)00542-7 12971900

[B12] Carlson J. R. O'gara B. A. (1983). The ecdysis of the cricket, *Teleogryllus oceanicus*: generation of the pharyngeal air swallowing motor program by the isolated frontal ganglion. Comp. Biochem. Physiol. 75, 579–587. 10.1016/0300-9629(83)90423-1

[B13] Chapman R. F. Ascoli-Christensen A. White P. R. (1991). Sensory coding for feeding deterrence in the grasshopper *Schistocerca americana*. J. Exp. Biol. 158, 241–259.

[B14] Chapman R. F. (1982). Chemoreception. The significance of receptor numbers. Adv. Insect Physiol. 16, 247–285. 10.1016/S0065-2806(08)60155-1

[B15] Chen T. W. Wardill T. J. Sun Y. Pulver S. R. Renninger S. L. Baohan A.. (2013). Ultrasensitive fluorescent proteins for imaging neuronal activity. Nature 499, 295–300. 10.1038/nature12354 23868258 PMC3777791

[B16] Chu B. Chui V. Mann K. Michael Gordon D. (2014). Presynaptic gain control drives sweet and bitter taste integration in *Drosophila*. Curr. Biol. 24, 1978–1984. 10.1016/j.cub.2014.07.020 25131672

[B17] Church R. B. Robertson F. W. (1966). Biochemical analysis of genetic differences in the growth of Drosophila. Genet. Res. 7, 383–407. 10.1017/S0016672300009836 5940873

[B18] Clifford M. N. (1985). Coffee: Botany, Biochemistry, and Production of Beans and Beverage. London; Westport, CN: Croom Helms; AVI Pub. Co.

[B19] Clyne P. J. Warr C. G. Carlson J. R. (2000). Candidate taste receptors in Drosophila. Science 287, 1830–1834. 10.1126/science.287.5459.1830 10710312

[B20] Cooper D. M. (1960). Food preferences of larval and adult Drosophila. Evolution 14, 41–55. 10.2307/2405921

[B21] Delventhal R. Carlson J. R. (2016). Bitter taste receptors confer diverse functions to neurons. eLife 5:e11181. 10.7554/eLife.11181 26880560 PMC4764594

[B22] Dethier V. G. (1978). Other tastes, other worlds. Science 201, 224–228. 10.1126/science.663651 663651

[B23] Dunipace L. Meister S. McNealy C. Amrein H. (2001). Spatially restricted expression of candidate taste receptors in the Drosophila gustatory system. Curr. Biol. 11, 822–835. 10.1016/S0960-9822(01)00258-5 11516643

[B24] El-Keredy A. Schleyer M. Konig C. Ekim A. Gerber B. (2012). Behavioural analyses of quinine processing in choice, feeding and learning of larval Drosophila. PLoS ONE 7:e40525. 10.1371/journal.pone.0040525 22802964 PMC3393658

[B25] Fishilevich E. Domingos A. I. Asahina K. Naef F. Vosshall L. B. Louis M. (2005). Chemotaxis behavior mediated by single larval olfactory neurons in Drosophila. Curr. Biol. 15, 2086–2096. 10.1016/j.cub.2005.11.016 16332533

[B26] Freeman E. G. Dahanukar A. (2015). Molecular neurobiology of Drosophila taste. Curr. Opin. Neurobiol. 34, 140–148. 10.1016/j.conb.2015.06.001 26102453 PMC4577450

[B27] French A. Ali Agha M. Mitra A. Yanagawa A. Sellier M. J. Marion-Poll F. (2015). Drosophila bitter taste(s). Front. Integr. Neurosci. 9:58. 10.3389/fnint.2015.00058 26635553 PMC4658422

[B28] Gendre N. Luer K. Friche S. Grillenzoni N. Ramaekers A. Technau G. M.. (2004). Integration of complex larval chemosensory organs into the adult nervous system of Drosophila. Development 131, 83–92. 10.1242/dev.00879 14645122

[B29] Gerber B. Hendel T. (2006). Outcome expectations drive learned behaviour in larval Drosophila. Proc. Biol. Sci. 273, 2965–2968. 10.1098/rspb.2006.3673 17015355 PMC1639518

[B30] Gordon M. D. Scott K. (2009). Motor control in a drosophila taste circuit. Neuron 61, 373–384. 10.1016/j.neuron.2008.12.033 19217375 PMC2650400

[B31] Griss C. Simpson S. J. Rohrbacher J. Rowell C. H. F. (1991). Localization in the central nervous system of larval *Manduca-sexta* (Lepidoptera, Sphingidae) of areas responsible for aspects of feeding-behavior. J. Insect Physiol. 37, 477–482. 10.1016/0022-1910(91)90023-S

[B32] Haskell P. T. Schoonhoven L. M. (1969). The function of certain mouth part receptors in relation to feeding in *Schistocerca Gregaria* and *Locusta Migratoria* Migratorioides. Entomol. Exp. Appl. 12, 423–440. 10.1111/j.1570-7458.1969.tb02538.x

[B33] Hergarden A. C. Tayler T. D. Anderson D. J. (2012). Allatostatin-A neurons inhibit feeding behavior in adult Drosophila. Proc. Natl. Acad. Sci. U.S.A. 109, 3967–3972. 10.1073/pnas.1200778109 22345563 PMC3309792

[B34] Hill L. Mordue W. Highnam K. C. (1966). The endocrine system, frontal ganglion, and feeding during maturation in the female desert locust. J. Insect Physiol. 12, 1197–1208. 10.1016/0022-1910(66)90132-6 6004926

[B35] Huser A. Rohwedder A. Apostolopoulou A. A. Widmann A. Pfitzenmaier J. E. Maiolo E. M.. (2012). The serotonergic central nervous system of the Drosophila larva: anatomy and behavioral function. PLoS ONE 7:e47518. 10.1371/journal.pone.0047518 23082175 PMC3474743

[B36] Ito K. Suzuki K. Estes P. Ramaswami M. Yamamoto D. Strausfeld N. J. (1998). The organization of extrinsic neurons and their implications in the functional roles of the mushroom bodies in *Drosophila melanogaster* Meigen. Learn. Mem. 5, 52–77. 10454372 PMC311240

[B37] Jiao Y. Moon S. J. Wang X. Ren Q. Montell C. (2008). Gr64f is required in combination with other gustatory receptors for sugar detection in Drosophila. Curr. Biol. 18, 1797–1801. 10.1016/j.cub.2008.10.009 19026541 PMC2676565

[B38] Joseph R. M. Carlson J. R. (2015). Drosophila chemoreceptors: a molecular interface between the chemical world and the brain. Trends Genet. 31, 683–695. 10.1016/j.tig.2015.09.005 26477743 PMC4674303

[B39] Kikut-Ligaj D. Trzcielinska-Lorych J. (2015). How taste works: cells, receptors and gustatory perception. Cell. Mol. Biol. Lett. 20, 699–716. 10.1515/cmble-2015-0042 26447485

[B40] Kim H. Choi M. S. Kang K. Kwon J. Y. (2016). Behavioral analysis of bitter taste perception in Drosophila larvae. Chem. Senses 41, 85–94. 10.1093/chemse/bjv061 26512069

[B41] Klein M. Afonso B. Vonner A. J. Hernandez-Nunez L. Berck M. Tabone C. J.. (2015). Sensory determinants of behavioral dynamics in *Drosophila thermotaxis*. Proc. Natl. Acad. Sci. U.S.A. 112, E220–E229. 10.1073/pnas.1416212112 25550513 PMC4299240

[B42] König C. Schleyer M. Leibiger J. El-Keredy A. Gerber B. (2014). Bitter-sweet processing in larval Drosophila. Chem. Senses 39, 489–505. 10.1093/chemse/bju016 24833133

[B43] Kreher S. A. Kwon J. Y. Carlson J. R. (2005). The molecular basis of odor coding in the Drosophila larva. Neuron 46, 445–456. 10.1016/j.neuron.2005.04.007 15882644

[B44] Kurada P. White K. (1998). Ras promotes cell survival in Drosophila by downregulating hid expression. Cell 95, 319–329. 10.1016/S0092-8674(00)81764-X 9814703

[B45] Kwon J. Y. Dahanukar A. Weiss L. A. Carlson J. R. (2007). The molecular basis of CO_2_ reception in Drosophila. Proc. Natl. Acad. Sci. U.S.A. 104, 3574–3578. 10.1073/pnas.0700079104 17360684 PMC1805529

[B46] Kwon J. Y. Dahanukar A. Weiss L. A. Carlson J. R. (2011). Molecular and cellular organization of the taste system in the Drosophila larva. J. Neurosci. 31, 15300–15309. 10.1523/JNEUROSCI.3363-11.2011 22031876 PMC3225198

[B47] Kwon J. Y. Dahanukar A. Weiss L. A. Carlson J. R. (2014). A map of taste neuron projections in the Drosophila CNS. J. Biosci. 39, 565–574. 10.1007/s12038-014-9448-6 25116611 PMC4240268

[B48] Larsson M. C. Domingos A. I. Jones W. D. Chiappe M. E. Amrein H. Vosshall L. B. (2004). Or83b encodes a broadly expressed odorant receptor essential for Drosophila olfaction. Neuron 43, 703–714. 10.1016/j.neuron.2004.08.019 15339651

[B49] LeDue E. E. Chen Y. C. Jung A. Y. Dahanukar A. Gordon M. D. (2015). Pharyngeal sense organs drive robust sugar consumption in Drosophila. Nat. Commun. 6:6667. 10.1038/ncomms7667 25807033 PMC4375776

[B50] Lee Y. Kang M. J. Shim J. Cheong C. U. Moon S. J. Montell C. (2012). Gustatory receptors required for avoiding the insecticide L-canavanine. J. Neurosci. 32, 1429–1435. 10.1523/JNEUROSCI.4630-11.2012 22279227 PMC3356580

[B51] Lee Y. Kim S. H. Montell C. (2010). Avoiding DEET through insect gustatory receptors. Neuron 67, 555–561. 10.1016/j.neuron.2010.07.006 20797533 PMC2929391

[B52] Lee Y. Moon S. J. Montell C. (2009). Multiple gustatory receptors required for the caffeine response in Drosophila. Proc. Natl. Acad. Sci. U.S.A. 106, 4495–4500. 10.1073/pnas.0811744106 19246397 PMC2657413

[B53] Lee Y. Moon S. J. Wang Y. Montell C. (2015). A Drosophila gustatory receptor required for strychnine sensation. Chem. Senses 40, 525–533. 10.1093/chemse/bjv038 26187906 PMC4580539

[B54] Ma W. C. (1972). Dynamics of feeding responses in *Pieris brassicae* Linn. as a function of chemosensory input: a behavioral, ultrastructural and electrophysiological study. Meded. Lanbouwhogesch. Wageningen 72, 162.

[B55] Manzo A. Silies M. Gohl D. M. Scott K. (2012). Motor neurons controlling fluid ingestion in Drosophila. Proc. Natl. Acad. Sci. U.S.A. 109, 6307–6312. 10.1073/pnas.1120305109 22474379 PMC3341050

[B56] Marella S. Mann K. Scott K. (2012). Dopaminergic modulation of sucrose acceptance behavior in Drosophila. Neuron 73, 941–950. 10.1016/j.neuron.2011.12.032 22405204 PMC3310174

[B57] Menuz K. Larter N. K. Park J. Carlson J. R. (2014). An RNA-seq screen of the Drosophila antenna identifies a transporter necessary for ammonia detection. PLoS Genet. 10:e1004810. 10.1371/journal.pgen.1004810 25412082 PMC4238959

[B58] Meunier N. Marion-Poll F. J. Rospars P. Tanimura T. (2003). Peripheral coding of bitter taste in Drosophila. J. Neurobiol. 56, 139–152. 10.1002/neu.10235 12838579

[B59] Milan N. F. Kacsoh B. Z. Schlenke T. A. (2012). Alcohol consumption as self-medication against blood-borne parasites in the fruit fly. Curr. Biol. 22, 488–493. 10.1016/j.cub.2012.01.045 22342747 PMC3311762

[B60] Miles C. I. Booker R. (1998). The role of the frontal ganglion in the feeding and eclosion behavior of the moth *Manduca sexta*. J. Exp. Biol. 201(Pt 11), 1785–1798. 9576889 10.1242/jeb.201.11.1785

[B61] Mishra D. Miyamoto T. Rezenom Y. H. Broussard A. Yavuz A. Slone J.. (2013). The molecular basis of sugar sensing in Drosophila larvae. Curr. Biol. 23, 1466–1471. 10.1016/j.cub.2013.06.028 23850280 PMC4294765

[B62] Moon S. J. Kottgen M. Jiao Y. Xu H. Montell C. (2006). A taste receptor required for the caffeine response *in vivo*. Curr. Biol. 16, 1812–1817. 10.1016/j.cub.2006.07.024 16979558

[B63] Moon S. J. Lee Y. Jiao Y. Montell C. (2009). A Drosophila gustatory receptor essential for aversive taste and inhibiting male-to-male courtship. Curr. Biol. 19, 1623–1627. 10.1016/j.cub.2009.07.061 19765987 PMC2762023

[B64] Mustard J. A. (2014). The buzz on caffeine in invertebrates: effects on behavior and molecular mechanisms. Cell. Mol. Life Sci. 71, 1375–1382. 10.1007/s00018-013-1497-8 24162934 PMC3961528

[B65] Ni L. Klein M. Svec K. V. Budelli G. Chang E. C. Ferrer A. J.. (2016). The Ionotropic Receptors IR21a and IR25a mediate cool sensing in Drosophila. eLife 5:e13254. 10.7554/eLife.13254 27126188 PMC4851551

[B66] Niewalda T. Singhal N. Fiala A. Saumweber T. Wegener S. Gerber B. (2008). Salt processing in larval Drosophila: choice, feeding, and learning shift from appetitive to aversive in a concentration-dependent way. Chem. Senses 33, 685–692. 10.1093/chemse/bjn037 18640967 PMC2565773

[B67] Oppliger F. Y. Guerin P. M. Vlimant M. (2000). Neurophysiological and behavioural evidence for an olfactory function for the dorsal organ and a gustatory one for the terminal organ in *Drosophila melanogaster* larvae. J. Insect Physiol. 46, 135–144. 10.1016/S0022-1910(99)00109-2 12770245

[B68] Pool A. H. Kvello P. Mann K. Cheung S. K. Gordon M. D. Wang L.. (2014). Four GABAergic interneurons impose feeding restraint in Drosophila. Neuron 83, 164–177. 10.1016/j.neuron.2014.05.006 24991960 PMC4092013

[B69] Pool A. H. Scott K. (2014). Feeding regulation in Drosophila. Curr. Opin. Neurobiol. 29C, 57–63. 10.1016/j.conb.2014.05.008 24937262 PMC4253568

[B70] Python F. Stocker R. F. (2002). Adult-like complexity of the larval antennal lobe of *D. melanogaster* despite markedly low numbers of odorant receptor neurons. J. Comp. Neurol. 445, 374–387. 10.1002/cne.10188 11920714

[B71] Robertson H. M. Warr C. G. Carlson J. R. (2003). Molecular evolution of the insect chemoreceptor gene superfamily in *Drosophila melanogaster*. Proc. Natl. Acad. Sci. U.S.A. 100(Suppl 2), 14537–14542. 10.1073/pnas.2335847100 14608037 PMC304115

[B72] Rohwedder A. Pfitzenmaier J. E. Ramsperger N. Apostolopoulou A. A. Widmann A. Thum A. S. (2012). Nutritional value-dependent and nutritional value-independent effects on *Drosophila melanogaster* larval behavior. Chem. Senses 37, 711–721. 10.1093/chemse/bjs055 22695795

[B73] Rohwedder A. Wenz N. L. Stehle B. Huser A. Yamagata N. Zlatic M.. (2016). Four individually identified paired dopamine neurons signal reward in Larval Drosophila. Curr. Biol. 26, 661–669. 10.1016/j.cub.2016.01.012 26877086

[B74] Schipanski A. Yarali A. Niewalda T. Gerber B. (2008). Behavioral analyses of sugar processing in choice, feeding, and learning in larval Drosophila. Chem. Senses 33, 563–573. 10.1093/chemse/bjn024 18511478 PMC2467463

[B75] Schleyer M. Saumweber T. Nahrendorf W. Fischer B. von Alpen D. Pauls D.. (2011). A behavior-based circuit model of how outcome expectations organize learned behavior in larval Drosophila. Learn. Mem. 18, 639–653. 10.1101/lm.2163411 21946956

[B76] Schoonhoven L. M. (1972). Some Aspects of Host Selection and Feeding in Phytophagous Insects. Amsterdam: North-Holland.

[B77] Schwarz S. Durisko Z. Dukas R. (2014). Food selection in larval fruit flies: dynamics and effects on larval development. Naturwissenschaften 101, 61–68. 10.1007/s00114-013-1129-z 24352256

[B78] Scott K. Brady R. Jr. Cravchik A. Morozov P. Rzhetsky A. Zuker C.. (2001). A chemosensory gene family encoding candidate gustatory and olfactory receptors in Drosophila. Cell 104, 661–673. 10.1016/S0092-8674(01)00263-X 11257221

[B79] Selcho M. Pauls D. Han K. A. Stocker R. F. Thum A. S. (2009). The role of dopamine in Drosophila larval classical olfactory conditioning. PLoS ONE 4:e5897. 10.1371/journal.pone.0005897 19521527 PMC2690826

[B80] Selcho M. Pauls D. Huser A. Stocker R. F. Thum A. S. (2014). Characterization of the octopaminergic and tyraminergic neurons in the central brain of Drosophila larvae. J. Comp. Neurol. 522, 3485–3500. 10.1002/cne.23616 24752702

[B81] Simpson S. J. James S. M. Simmonds S. J. Blaney W. M. (1991). Variation in chemosensitivity and the control of dietary selection behaviour in the locust. Appetite 17, 141–154. 10.1016/0195-6663(91)90069-5 1763906

[B82] Singh R. N. Singh K. (1984). Fine structure of the sensory organs of *Drosophila melanogaster* Meigen larva (Diptera: Drosophilidae). Int. J. Insect Morphol. Embryol. 13, 255–273. 10.1016/0020-7322(84)90001-1

[B83] Tissot M. Gendre N. Hawken A. Stortkuhl K. F. Stocker R. F. (1997). Larval chemosensory projections and invasion of adult afferents in the antennal lobe of Drosophila. J. Neurobiol. 32, 281–297. 9058321 10.1002/(sici)1097-4695(199703)32:3<281::aid-neu3>3.0.co;2-3

[B84] van Giesen L. Hernandez-Nunez L. Delasoie-Baranek S. Colombo M. Renaud P. Bruggmann R.. (2016). Multimodal stimulus coding by a gustatory sensory neuron in Drosophila larvae. Nat. Commun. 7:10687. 10.1038/ncomms10687 26864722 PMC4753250

[B85] Ventura A. K. Worobey J. (2013). Early influences on the development of food preferences. Curr. Biol. 23, R401–R408. 10.1016/j.cub.2013.02.037 23660363

[B86] Weiss L. A. Dahanukar A. Kwon J. Y. Banerjee D. Carlson J. R. (2011). The molecular and cellular basis of bitter taste in Drosophila. Neuron 69, 258–272. 10.1016/j.neuron.2011.01.001 21262465 PMC3033050

[B87] White K. Steller H. (1995). The control of apoptosis in Drosophila. Trends Cell Biol. 5, 74–78. 10.1016/S0962-8924(00)88950-3 14731416

[B88] White K. Tahaoglu E. Steller H. (1996). Cell killing by the Drosophila gene reaper. Science 271, 805–807. 10.1126/science.271.5250.805 8628996

[B89] Wintgens J. N. (2012). Coffee: Growing, Processing, Sustainable Production: A Guidebook for Growers, Processors, Traders, and Researchers. Weinheim: Wiley-VCH Verlag GmbH & Co.

[B90] Xu J. Sornborger A. T. Lee J. K. Shen P. (2008). Drosophila TRPA channel modulates sugar-stimulated neural excitation, avoidance and social response. Nat. Neurosci. 11, 676–682. 10.1038/nn.2119 18469811

[B91] Yapici N. Cohn R. Schusterreiter C. Ruta V. Vosshall L. B. (2016). A taste circuit that regulates ingestion by integrating food and hunger signals. Cell 165, 715–729. 10.1016/j.cell.2016.02.061 27040496 PMC5544016

[B92] Yong Jeong T. Shim J. So Oh R. Hong Yoon I. Chul Kim H. Seok Moon J.. (2013). An odorant-binding protein required for suppression of sweet taste by bitter chemicals. Neuron 79, 725–737. 10.1016/j.neuron.2013.06.025 23972598 PMC3753695

[B93] Zhang H. J. Anderson A. R. Trowell S. C. Luo A. R. Xiang Z. H. Xia Q. Y. (2011). Topological and functional characterization of an insect gustatory receptor. PLoS ONE 6:e24111. 10.1371/journal.pone.0024111 21912618 PMC3163651

[B94] Zhang Y. V. Ni J. Montell C. (2013). The molecular basis for attractive salt-taste coding in Drosophila. Science 340, 1334–1338. 10.1126/science.1234133 23766326 PMC4091975

