# Supplementary material for: Caffeine Taste Signaling in Drosophila Larvae
Source: Front Cell Neurosci. 2016 Aug 9;10:193. doi: 10.3389/fncel.2016.00193 (PMC4977282; doi:10.3389/fncel.2016.00193)
Supplement: Supplementary file 1 [file DataSheet1.docx]

**Caffeine taste signaling in *Drosophila* larvae**

**Anthi A. Apostolopoulou^1,2^, Saskia Köhn^1^, Bernhard Stehle^1^, Michael Lutz^1^, Alexander Wüst^1^, Lorena Mazija^1^, Anna Rist^1^, C. Giovanni Galizia^1,3^, Alja Lüdke^1^ and Andreas S. Thum^1,3*^**

^1^Department of Biology, University of Konstanz,

78464 Konstanz, Germany

^2^ Department of Biomedical Science, University of Sheffield,

S10 2TN Sheffield, United Kingdom

^3^Zukunftskolleg, University of Konstanz,

78464 Konstanz, Germany

***Correspondence**: Andreas S. Thum, Department of Biology, University of Konstanz,

Universitätsstraße 10, 78464 Konstanz, Germany

E-mail: andreas.thum@uni-konstanz.de

Running head: Caffeine in *Drosophila* larvae

**Supplemental methods:**

**Fly stocks and maintenance**

Flies were maintained on standard *Drosophila* medium at 25°C. For all experiments, flies were transferred to new vials and allowed to lay eggs for two days. Experiments were performed five or six days after egg laying. 3rd instar, feeding stage larvae were used in groups of about 30 animals for behavioral experiments or individually for anatomical approaches and Ca^2+^-imaging experiments. Orco mutants (Or83b) were obtained from the Bloomington Stock Center (23130). All other transgenic lines are described in the Materials and Methods section.

**Feeding**

Experiments were performed using standard methods [1; 2; 3; 4; 5]. Further details are given in the Materials and Methods section. To analyze if the agarose concentration changes feeding we expanded our approach by testing agarose concentrations of 1%, 2.5%, 3%, and 3.5% (w/v).

**Odor-Caffeine Learning**

Experiments were performed using standard methods [1; 2; 4; 5]. Further details are given in the Materials and Methods. To analyze if the agarose concentration changes odor-caffeine learning we expanded our analysis by testing agarose concentrations of 1%, 2.5%, 3%, and 3.5% (w/v).

**Olfactory choice behavior**

Experiments were performed using standard methods [1; 6]. 1.0% (w/v) agarose solution (Sigma Aldrich Cat. No.: A5093; CAS No.: 9012-36-6) was boiled in a microwave and filled as a thin layer into Petri dishes (85 mm diameter, Cat. No.: 82.1472, Sarstedt, Nümbrecht, Germany). As olfactory stimuli we either used 10 μl amyl acetate (AM, Fluka cat. no.: 46022; diluted 1:50 in paraffin oil, Fluka cat. no.: 76235), 10 µl 3-octanol (OCT, undiluted; Fluka cat. no.: 74850), or 10 µl 50 mM caffeine solution (Sigma Aldrich cat. no.: 27600). The odorants were loaded into custom-made Teflon containers (4.5-mm diameter) with perforated lids as described in Scherer et al. (2003). For the test, 30 larvae were put in the middle of a Petri dish. Larvae were counted after 5 minutes as being located on either the odor side, the no-odor side, or a middle neutral side (an area of about 10 mm width running vertically in the middle of the plate).

The preference indices for were calculated as follows:

Preference Index = (# ODOR - # NO ODOR ) / # TOTAL

Positive preference indices therefore indicate attraction of the stimulus.

**Statistical Methods**

Kruskal-Wallis tests were performed and, in case of significance, followed by Wilcoxon rank-sum tests; Holm-Bonferroni corrections were used for multiple comparisons as applicable. Likewise, Wilcoxon signed-ranked tests were used to compare values against chance level. All statistical analyses were performed with R version 2.14.0 and Windows Excel 2010. Figure alignments were done with Adobe Photoshop. The behavioral data are presented as boxplots (middle line, median; box boundaries, 25% / 75% quantiles; whiskers, 10% / 90% quantiles; circles, outliers). Asterisks (*, **, ***) and ‘‘n.s.’’ indicate p < 0.05, p < 0.01, p < 0.001, and p > 0.05, respectively.

**Light microscopy**

Dissection of 3rd instar larvae was performed in phosphate-buffered saline (PBS). After fixation in 3.7% formaldehyde (Merck, Darmstadt, Germany) in PBS for 30 min, brains or heads were washed seven times in PBT (PBS with 3% Triton-X 100, Sigma-Aldrich, St. Louis, MO). Next, 5% normal goat serum (Vector Laboratories, Burlingame, CA) in PBT was added for 2 h. Primary antibodies were applied for 2 days at 4°C. Samples were then washed six times with PBT. Secondary antibodies were applied for 2 days at 4°C and specimens were washed eight times with PBT. Finally, samples were mounted in Vectashield (Vector Laboratories, Burlingame, CA) between two cover slips and stored at 4°C in the dark.

Anti-GFP [Anti-GFP Rabbit, polyclonal serum, A6455, Molecular Probes, (Eugene, OR), 1:1000] was used to label the Grs expression in the SOG or the periphery. Anti-ChAT [ChAT4B1 Mouse, monoclonal ChAT4B1, DSHB (Iowa City, IA)1:100] was applied to label the neuropil, whereas anti-FasII [1D4 anti-Fasciclin II Mouse, monoclonal, 1D4, DSHB (Iowa City, IA) 1:50] was used to label the axonal tracts of the SOG. Anti-elav [Anti-elav mouse, DHSB (Iowa City, IA), 1:100] served to visualize neuronal nuclei in the periphery. As secondary antibodies, IgG Alexa Fluor 488 [goat anti-rabbit IgG Alexa Fluor 488, A11008; Molecular Probes, 1:200] and IgG Alexa Fluor 647 [goat anti-mouse IgG Alexa Fluor 647 A21236; Molecular Probes, 1:200] were used. Images were obtained using a Zeiss LSM510 confocal microscope (Zeiss, Jena, Germany) with a 25-fold oil immersion objective. Image stacks were projected and analyzed with Image J software (http://imagej.nih.gov/ij). Photoshop (Adobe Systems Inc., San José, CA) was used for contrast and brightness adjustment as well as for rotation and organization of the images.

**Supporting Information**

**Supplemental Figure 1: The amount of consumed caffeine containing substrate correlates with its reinforcing potential**

(A) Caffeine-dependent feeding shows a tendency to increase with higher agarose concentrations up to 3% (the 3^rd^ boxplot is the same as shown in Figure 1C). At an agarose concentration of 3.5% caffeine-dependent feeding slightly decreases when compared with feeding rates at 3%. However, none of the described effects is significantly different after statistical evaluation (p=0.2995). (B) Caffeine-dependent learning shows a similar tendency to increase with higher agarose concentrations up to 3%. Again, learning decreases by further increasing the agarose concentration to 3.5% (for 1 % p=0.7078, for 2.5 % p=0.0212, for 3 % p=0.0097, for 3.5 % p=0.0070, when learning was compared against random distribution). (C) The data suggests for a positive correlation of caffeine-dependent feeding and learning. Significances against a mean of 0 are shown below each box-plot in A and B. Differences among experimental groups are depicted above the respective box plots in A and B. n.s. non-significant p > 0.05, * p < 0.05 and ** p < 0.01. Sample size for each group n ≥ 12. Small circles indicate outliers.

**Supplemental Figure 2:** **Expression patterns of the different Gr-Gal4 drivers.** (A-G) The left panels show a schematic overview of the expression patterns of each Gal4 driver in the sets of six bitter neurons of the external TO (B1, B2 and C1-4) and six bitter neurons of the pharyngeal DPS (D1 and D2), VPS (E1 and E2) and PPS (F1 and F2). On the right side the expression patterns of each Gal4 driver crossed with UAS-*mCD8::GFP* (GFP staining in green, anti-elav cell body staining in magenta) are shown. The four panels always show TOG/DOG, DPS, VPS, and PPS (from the top left to the bottom right). In general, we observe similar anatomical results as published before. Whereas *Gr66a-Gal4* and *Gr33a-Gal4* label all twelve bitter GRNs (A and B), all other driver lines show specific expression in only a single GRN: *Gr10a-Gal4* in B2 (C), *Gr36c-Gal4* in C1 (D), *Gr94a-Gal4* in C2 (E), *Gr97a-Gal4* in C3 (F) and *Gr93a-Gal4* in D1 (G). In addition, *Gr93a-Gal4* shows expression in two olfactory receptor neurons (in G left top panel and H, ORN). (H) Frontal view of a partial z-projection of the brain hemispheres and the SOG of a *Gr93a-Gal4/UAS-mCD8::GFP* larval brain. The expression pattern of *Gr93a-Gal4* is visualized by anti-GFP antibody staining (green). Anti-ChAT/anti-FasII antibody staining is used as neuropil marker (magenta). Axon terminals of two ORNs in the antennal lobe (arrows ORN) and a single GRN in the SOG (arrowheads) are visible. Scale bars: 25 µm.

**Supplemental Figure 3: Ablation of the pharyngeal D1 neuron pair does not abolish quinine-dependent choice behavior**

(A) Schematic organization of the set of 12 bitter GRNs that is used to summarize the Gal4 line expression pattern. Depicted are the external terminal organ (TO), and internal gustatory organs (DPS: dorsal pharyngeal sense organ, VPS: ventral pharyngeal sense organ, PPS: posterior pharyngeal sense organ). (B) Quinine choice behavior of larvae lacking the D1 GRN pair (p=2*10^-4^ against chance levels, p=0.0870 compared to the Gal4 control and p=0.0150 compared to the UAS*-hid,rpr* control). The data shows that larvae lacking the D1 GRN pair are still able to avoid 5 mM of quinine. Control genotypes are shown in gray, experimental groups at the center in red. Sample size for each group n ≥ 14; Significances against a mean of 0 are given at the bottom in B. Significances between experimental groups are depicted above the respective box plots in B. n.s. non-significant p > 0.05, * p < 0.05 and *** p < 0.001.

**Supplemental Figure 4: Caffeine is not perceived by the larval olfactory sensory system**

(A) Larvae do not show any olfactory preference for caffeine when presenting it in Teflon containers to avoid direct contact (n=12; p=0.9374). (B) Orco mutants show a similar gustatory preference towards caffeine compared to wild type control larvae. The data suggest that caffeine is not sensed by the olfactory sensory system (n=10 for wild type and n=9 for or83b-/-; p=0.1910). Significances against a mean of 0 are shown below each box-plot in A and B. Differences between experimental groups are depicted in B above the respective box plots. n.s., non-significant p > 0.05, * p < 0.05, and ** p < 0.01. Small circles indicate outliers.

**Supplemental Figure 5: Odor acuity towards AM and OCT remains intact after ablation of Gr33a- or Gr93a-Gal4 positive GRNs**

(A-B) Ablation of *Gr33a-Gal4* or *Gr93a-Gal4* positive GRNs does not change the innate response of experimental larvae towards the odor AM (p=0.0015 and p=0.0020 respectively). (C-D) Ablation of *Gr33a-Gal4*- or *Gr93a-Gal4*-positive GRNs does not change the innate response of experimental larvae towards the odor OCT (p=0.0006 and p=8*10^-5^ respectively). Sample size for each group is n ≥ 12. Significances against a mean of 0 are shown at the bottom of each panel. Differences among groups are depicted below the respective box plots. n.s. non-significant p > 0.05, ** p < 0.01 and *** p < 0.001. Small circles indicate outliers.

**Supplemental Figure 6:** **Ectopic expression of the *Gr93a* receptor gene in the pharyngeal D2 neuron does not elicit a response upon caffeine stimulation.**

(A) Calcium increase upon 25 mM caffeine stimulation was recorded in *Gr33a-Gal4*; UAS*-Gr93a;* UAS-*GCaMP6m* experimental animals. Left panels depict the raw fluorescence images before and during caffeine application (25 mM). At the center, the same pictures are shown including the selection of three ROIs that mark the D1 neuron, the D2 neuron in its close proximity and a control ROI in similar proximity. This allows us to control for an artificial fluorescence increase in the D2 cell that originates from D1 cell fluorescence. The rightmost panels show the ΔF/F false color coded images that depict the fluorescent increase of the D1 cell during caffeine application.

**References**

[1] A.A. Apostolopoulou, L. Mazija, A. Wust, and A.S. Thum, The neuronal and molecular basis of quinine-dependent bitter taste signaling in Drosophila larvae. Frontiers in behavioral neuroscience 8 (2014) 6.

[2] A. El-Keredy, M. Schleyer, C. Konig, A. Ekim, and B. Gerber, Behavioural analyses of quinine processing in choice, feeding and learning of larval Drosophila. Plos One 7 (2012) e40525.

[3] T. Niewalda, N. Singhal, A. Fiala, T. Saumweber, S. Wegener, and B. Gerber, Salt processing in larval Drosophila: choice, feeding, and learning shift from appetitive to aversive in a concentration-dependent way. Chem Senses 33 (2008) 685-92.

[4] A. Rohwedder, J.E. Pfitzenmaier, N. Ramsperger, A.A. Apostolopoulou, A. Widmann, and A.S. Thum, Nutritional value-dependent and nutritional value-independent effects on Drosophila melanogaster larval behavior. Chem Senses 37 (2012) 711-21.

[5] A. Schipanski, A. Yarali, T. Niewalda, and B. Gerber, Behavioral analyses of sugar processing in choice, feeding, and learning in larval Drosophila. Chem Senses 33 (2008) 563-73.

[6] A.A. Apostolopoulou, A. Widmann, A. Rohwedder, J.E. Pfitzenmaier, and A.S. Thum, Appetitive associative olfactory learning in Drosophila larvae. Journal of visualized experiments : JoVE (2013).
