# Supplementary figures and images for: Caffeine Taste Signaling in Drosophila Larvae
Source: Front Cell Neurosci. 2016 Aug 9;10:193. doi: 10.3389/fncel.2016.00193 (PMC4977282; doi:10.3389/fncel.2016.00193)

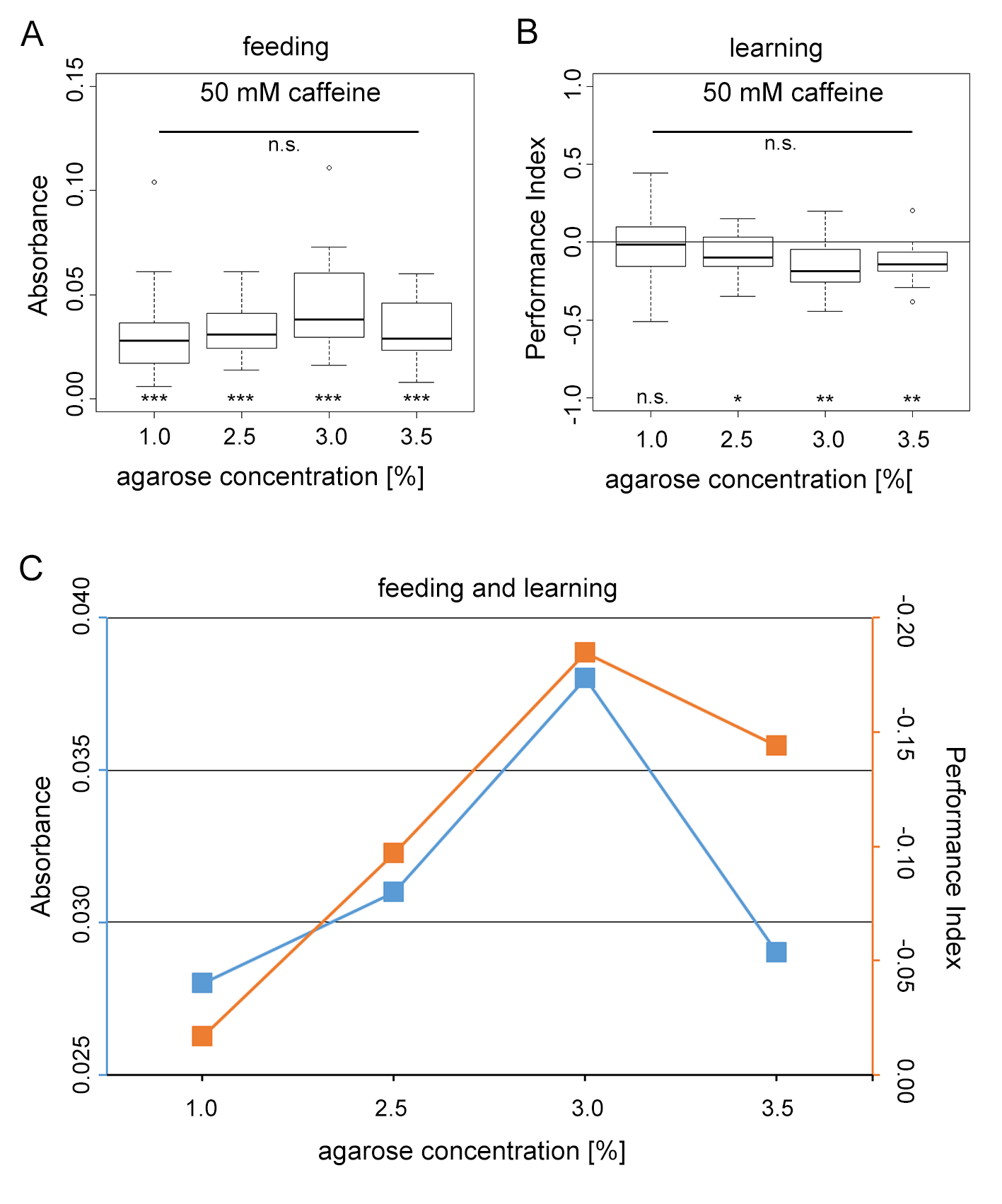

Supplement: Supplementary file 2 [file Image1.TIF]

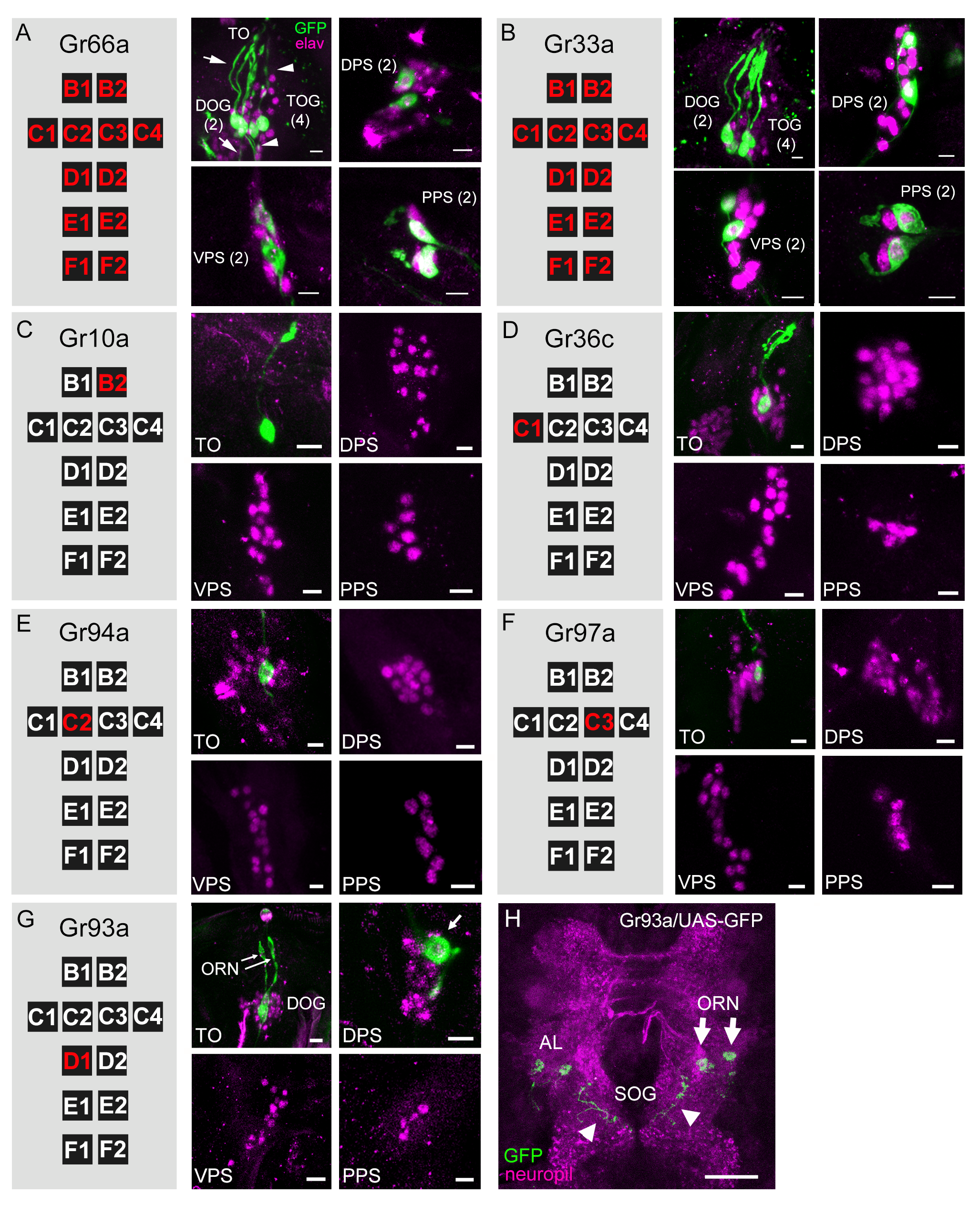

Supplement: Supplementary file 3 [file Image2.TIF]

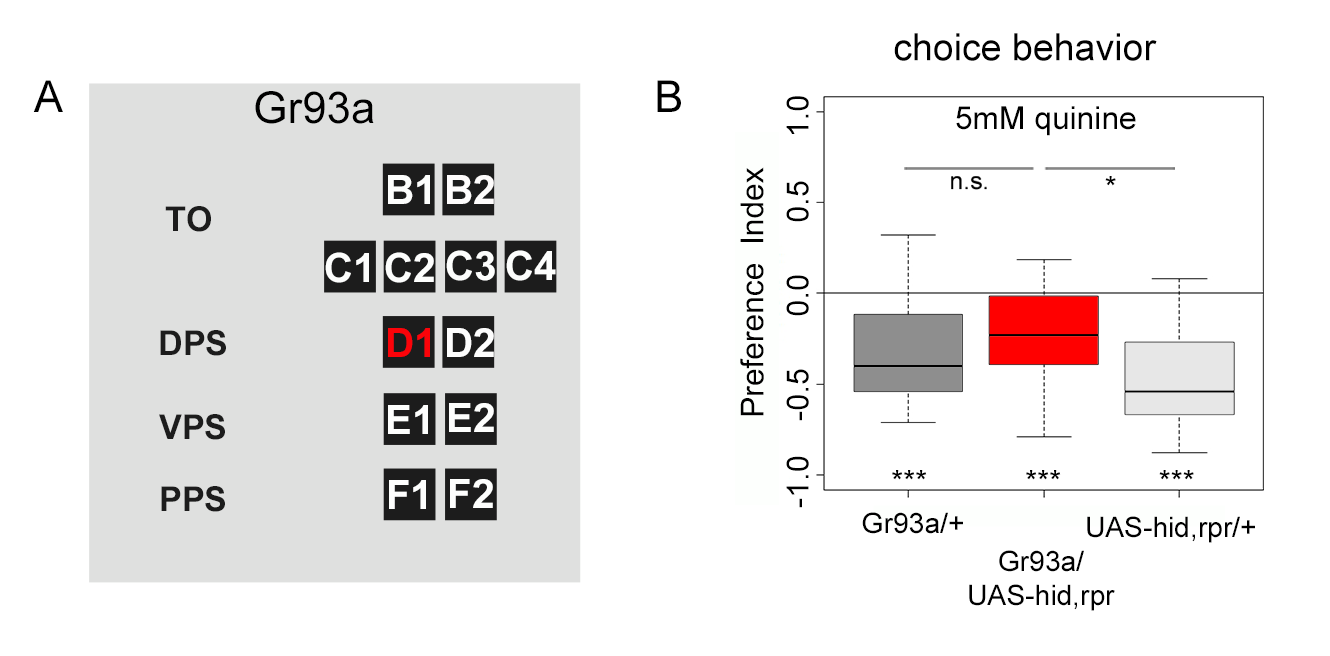

Supplement: Supplementary file 4 [file Image3.TIF]

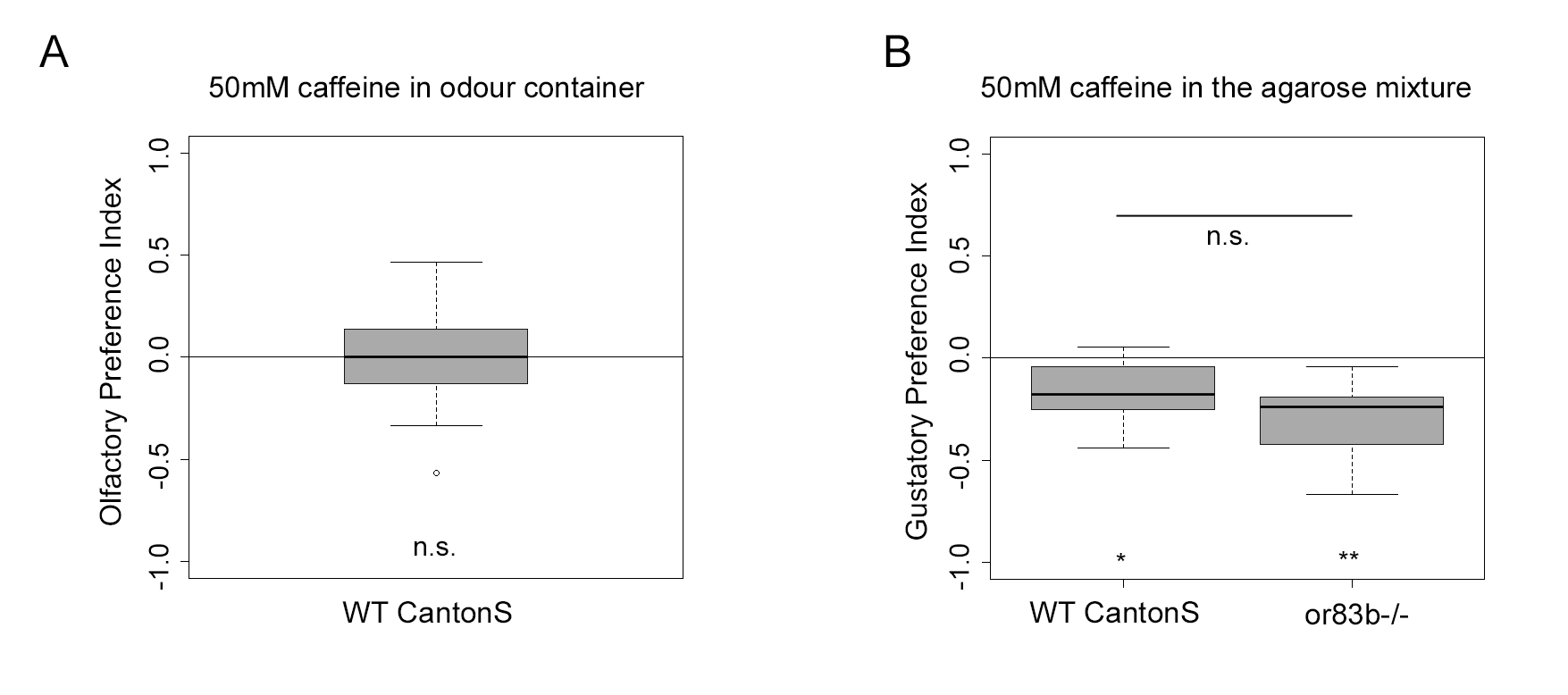

Supplement: Supplementary file 5 [file Image4.TIF]

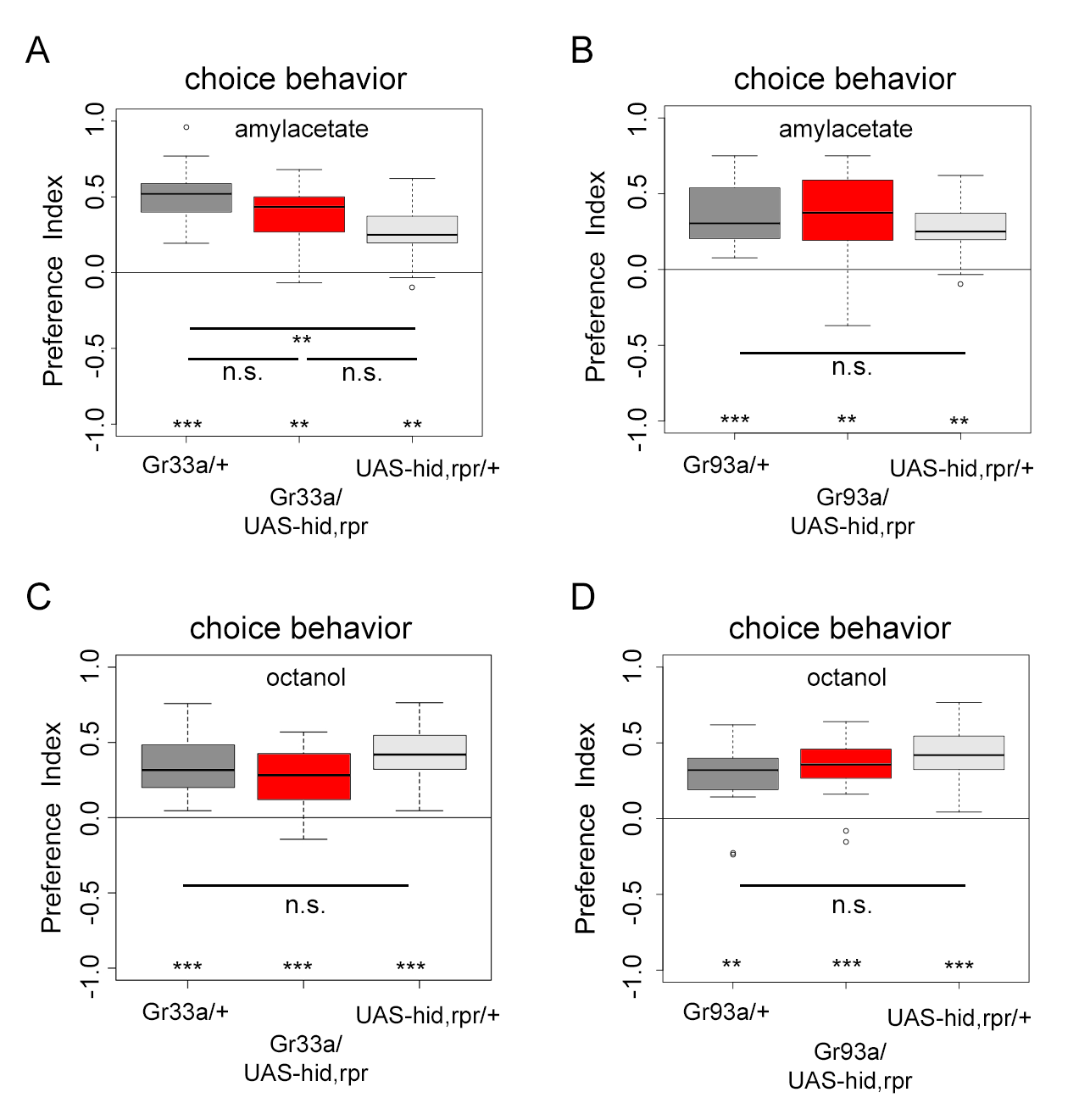

Supplement: Supplementary file 6 [file Image5.TIF]

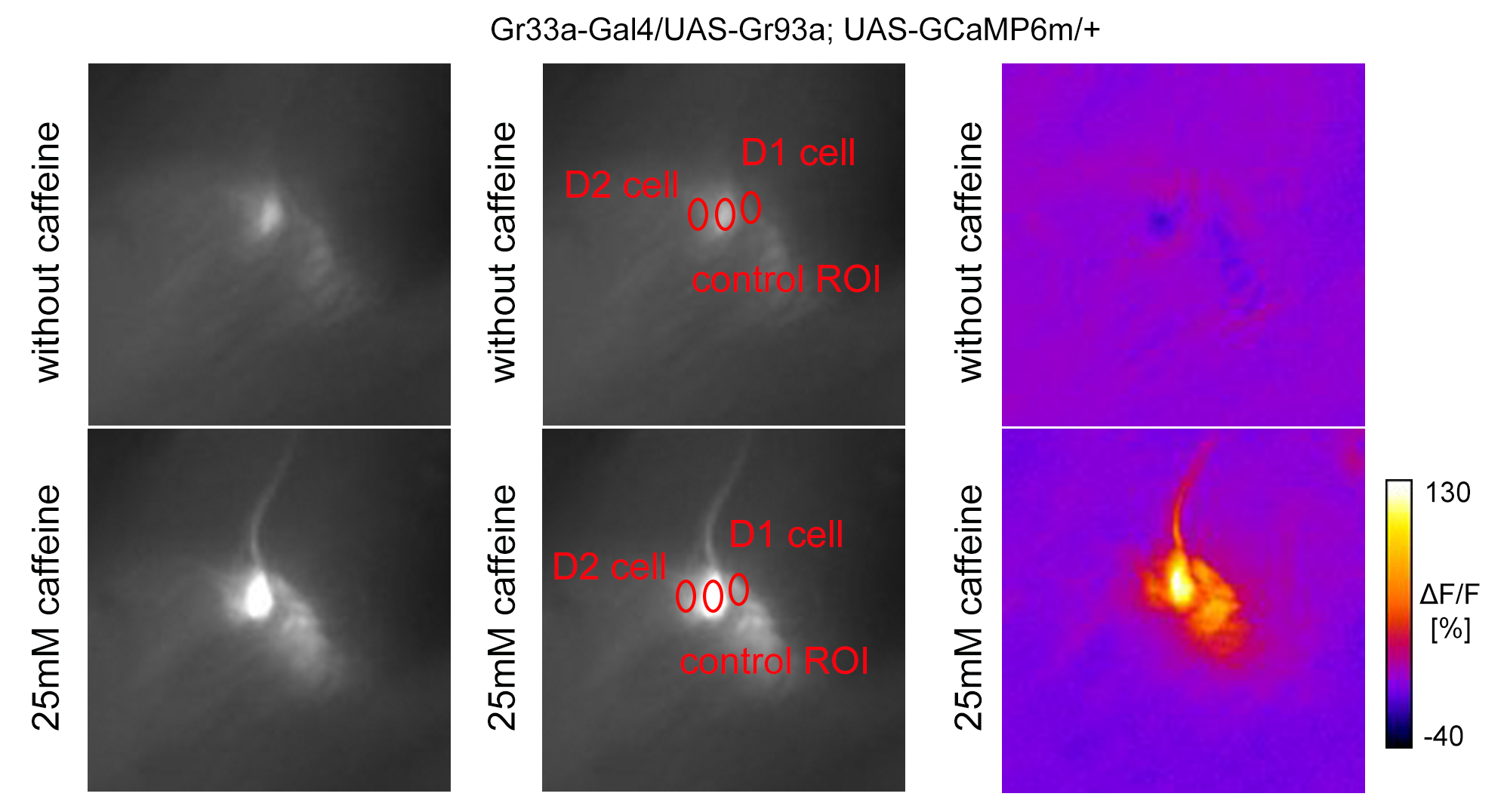

Supplement: Supplementary file 7 [file Image6.TIF]
